# Genomic and Transcriptomic Analysis of High-Grade Endometrial Carcinoma Reveals Biological Heterogeneity and Molecular Classification Challenges

**DOI:** 10.1158/2767-9764.CRC-25-0589

**Published:** 2026-04-28

**Authors:** Masahito Kawazu, Ayumi Taguchi, Emiko Yoshida, Hiroshi Yoshida, Masaya Uno, Satoshi Inoue, Yoko Yamamoto, Shingo Sakashita, Toshihide Ueno, Yuki Nakamura, Jason Lin, Shinya Kojima, Katsushige Kawase, Aya Ishizaka, Suguru Miyata, Motohiro Kojima, Masako Ikemura, Kenbun Sone, Mitsuya Ishikawa, Tomoyasu Kato, Hiroyuki Mano, Yasuhisa Terao, Katsutoshi Oda

**Affiliations:** 1 Division of Cell Therapy, Chiba Cancer Center Research Institute, Chiba, Japan.; 2 Division of Cellular Signaling, National Cancer Center Research Institute, Tokyo, Japan.; 3 Department of Obstetrics and Gynecology, Graduate School of Medicine, The University of Tokyo, Tokyo, Japan.; 4 Department of Obstetrics and Gynecology, Juntendo University Faculty of Medicine, Tokyo, Japan.; 5 Diagnosis and Therapeutics of Intractable Diseases and Intractable Disease Research Center, Juntendo University Graduate School of Medicine, Tokyo, Japan.; 6 Department of Diagnostic Pathology, National Cancer Center Hospital, Tokyo, Japan.; 7 Department of Gynecology, National Cancer Center Hospital, Tokyo, Japan.; 8 Division of Tumor Immunology, Institute for Advanced Medical Research, Keio University School of Medicine, Tokyo, Japan.; 9 Division of Pathology, Exploratory Oncology Research and Clinical Trial Center, National Cancer Center Japan, Tokyo, Japan.; 10 Department of Otorhinolaryngology/Head & Neck Surgery, Graduate School of Medicine, Chiba University, Chiba, Japan.; 11 Department of Pathology, Graduate School of Medicine, The University of Tokyo, Tokyo, Japan.; 12 Division of Integrative Genomics, Graduate School of Medicine, The University of Tokyo, Tokyo, Japan.

## Abstract

High-grade endometrial carcinoma exhibits marked histologic diversity, yet its molecular basis and the potential contribution of transcriptomic phenotyping to molecular classification remain incompletely understood. To address this, we performed whole-exome sequencing and RNA sequencing on 81 high-grade endometrial carcinomas, including serous, clear cell, grade 3 endometrioid, and carcinosarcoma. Tumors were assigned to molecular subtypes (*POLE*-ultramutated, microsatellite instability–high (MSI-H), *TP53*-mutated (*TP53*-mut), and no specific molecular profile (NSMP), based on The Cancer Genome Atlas and ProMisE frameworks. Transcriptomic phenotypes were identified by unsupervised clustering of gene expression and analyzed in relation to histology, molecular subtypes, immune-related gene expression, and clinical outcomes. In this context, substantial discordance was observed among *TP53* mutation status, p53 immunohistochemistry (IHC), and copy number–based classification in non-*POLE*/non–MSI-H tumors. Transcriptomic clustering identified three phenotypic groups linked to cell differentiation status: glandular/luminal, ciliated, and epithelial–mesenchymal transition-like (EMT-like). These phenotypes transcended molecular subtype boundaries. For example, *TP53*-mut tumors were distributed across both glandular/luminal and EMT-like phenotypes. The glandular/luminal phenotype was associated with elevated antigen presentation (e.g., HLA expression) and immune-related signaling, whereas the EMT-like phenotype, frequently observed in carcinosarcoma, was linked to stemness and metastatic potential. *TP53*-mut and NSMP were associated with poor prognosis in high-grade endometrial carcinoma, whereas the glandular/luminal phenotype was associated with better outcomes than the EMT-like phenotype, an effect largely influenced by carcinosarcoma prevalence. Transcriptomic phenotypes complement molecular subtypes in high-grade endometrial carcinoma, enhancing biological resolution and capturing clinically relevant heterogeneity. These results underscore persistent challenges of current molecular classification approaches, supporting the need for integrative strategies in high-grade disease.

**Significance:**

High-grade endometrial carcinomas show marked heterogeneity. We demonstrate substantial discordance among *TP53* mutation status, p53 IHC, and copy number–based assignment, alongside transcriptomic phenotypes spanning molecular subtypes. These findings provide a foundation for developing improved classification approaches to better interpret the heterogeneous molecular landscape of high-grade disease.

## Introduction

The incidence of endometrial carcinoma has been increasing worldwide, including among younger populations (1). Traditionally, endometrial carcinoma has been classified into two types: type I and type II tumors based on clinicopathologic features. However, recent genomic studies have led to a refined classification into four molecular subtypes. Based on genomic analysis by The Cancer Genome Atlas (TCGA), four subtypes included *POLE*-ultramutated, microsatellite instability–high (MSI-H), copy number–high (CN-H, typically *TP53*-mutated), and copy number–low (CN-L; ref. 2). The ProMisE classifier facilitated its clinical application by using immunohistochemistry (IHC) for mismatch repair (MMR) proteins and p53 and categorized *POLE*-mut, MMR-deficient (MMRd), p53 wild-type (WT), and p53 abnormal (p53-abn; refs. 3–5). Based on these frameworks, molecular classification has been increasingly incorporated into endometrial carcinoma management and was adopted in the 2020 World Health Organization (WHO) classification (6) and the updated 2023 FIGO staging system (7).

Nevertheless, a significant challenge remains; the correlation between molecular subtypes and histologic classification is weak, particularly in high-grade endometrial carcinomas (5). For example, clear cell and carcinosarcoma subtypes encompass all four molecular classes (8, 9). No specific molecular profile (NSMP), which is common in type I endometrial carcinomas, also appears in high-grade tumors (10, 11), but its clinical significance in this context remains poorly defined. Importantly, the prognostic behavior of NSMP tumors in high-grade endometrial carcinoma has not been adequately clarified, raising concerns about the consistency and precision of current molecular classification frameworks, which were largely established based on low-grade endometrial carcinomas and may not be directly applicable to high-grade disease.

Immune checkpoint inhibitors (ICI) have shown substantial efficacy as monotherapy in patients with MSI-H or MMRd endometrial carcinoma in the KEYNOTE-158 study (12). The combination of lenvatinib and pembrolizumab has been approved based on the KEYNOTE-775 trial (13), as a treatment option for patients without MMRd (i.e., MMR-proficient; pMMR), although the benefit was more pronounced in MMRd patients. In parallel, the clinical landscape of endometrial carcinoma treatment is shifting. Recent trials, including NRG-GY018 (pembrolizumab), RUBY (dostarlimab), DUO-E (durvalumab), and AtTEnd (atezolizumab), have demonstrated the clinical benefit of combining ICIs with chemotherapy in patients with endometrial carcinoma (14–17). These trials showed pronounced benefit in MMRd or MSI-H tumors but also demonstrated a statistically significant, albeit more modest, progression-free survival (PFS) improvement in pMMR subgroups. As these therapies carry significant financial and toxicity burdens, the development of reliable biomarkers to identify patients most likely to benefit from ICIs, particularly beyond MSI-H status, is a pressing need.

Recent studies have suggested that immune response in endometrial carcinoma may be influenced by factors beyond MSI-H status, including specific gene alterations such as *ARID1A* and tumor-infiltrating lymphocyte profiles (18–20). Yet, the association between immune characteristics, molecular subtypes, and tumor phenotype remains insufficiently understood. In addition, the cellular origin of high-grade endometrial carcinoma is still unclear. The endometrial epithelium is composed of ciliated and secretory cells, spatially organized into luminal and glandular layers, which dynamically change across the menstrual cycle. Single-cell and spatial transcriptomic studies have begun to map this landscape (21–25), but it is unknown how these epithelial lineages relate to endometrial carcinoma subtypes or prognosis.

To address these gaps, we investigated whether transcriptome-based phenotypic classification of high-grade endometrial carcinomas can provide insights about cellular origin and immune status. This study seeks to determine whether phenotypic stratification can refine risk assessment and potentially inform therapeutic strategies, particularly in molecularly ambiguous subtypes such as NSMP and p53-abn.

## Materials and Methods

### Patients and pathologic review

An overview of the sample processing and analytic workflow of this study is provided in Supplementary Fig. S1. We analyzed 81 tumors classically categorized as type II, encompassing nonendometrioid tumors as well as grade 3 endometrioid carcinomas (EMG3). Pathologic diagnoses were initially made according to the 2020 WHO classification (https://tumourclassification.iarc.who.int/welcome/; ref. 6) by at least two pathologists at each institution. Subsequently, a gynecologic pathologist (H. Yoshida) conducted central review to confirm histologic type. The analyzed tumors included 16 samples of serous carcinoma, 23 samples of clear cell carcinoma, 21 samples of carcinosarcoma, 19 samples of EMG3, and 2 other samples (including 1 small cell carcinoma and 1 unclassifiable poorly differentiated carcinoma; Supplementary Table S1). Among clear cell carcinomas, seven tumors were of mixed type containing endometrioid carcinoma components and two tumors were of mixed type containing serous components. Mixed carcinoma was defined as a carcinoma composed of two or more discrete histologic types of endometrial carcinoma, with at least one component being serous or clear cell carcinoma. Carcinosarcoma and dedifferentiated carcinoma were not included in this category. In line with WHO criteria, no quantitative dominant-component threshold (e.g., ≥50%) was applied (6). Eight MSI-H tumors of grade 1 to 2 endometrioid carcinoma were also analyzed. Representative histologic images are shown in Supplementary Fig. S2A–S2F. All pathologists were blinded to genomic analysis results and prognostic information.

This study was designed as an exploratory analysis of archived high-grade endometrial carcinoma specimens (*n* = 81). Although a formal power analysis was not performed, the sample size was determined based on expected molecular subtype distributions. Previous studies have shown that in high-grade endometrial carcinoma, the relative frequencies of molecular subtypes are approximately *POLE* (∼10%), MSI-H (∼20%), *TP53*-abn (∼40 to 50%), and NSMP (∼20 to 30%). Thus, a cohort of around 80 cases was considered sufficient to include a representative number of tumors in each subtype for comparative analyses (26, 27).

All surgical specimens were fixed according to the standard protocol of each participating institution, with fixation performed in neutral buffered formalin (10% or 20%) for 12 to 48 hours following the collection of frozen tissue samples. Frozen tumor samples were used for genomic analysis. Formalin-fixed paraffin-embedded sections, including representative tumor areas, were provided for IHC analysis. Clinicopathologic data for each patient were retrospectively obtained from medical records.

This study was conducted in accordance with the Declaration of Helsinki and approved by the ethics committee of the Chiba Cancer Center (approval number: M04-001). Written informed consent for research use of clinical information and tissue samples was obtained from all participants. Patients with high-grade endometrial carcinoma who underwent surgical resection at Juntendo University (2013–2023), The University of Tokyo Hospital (2010–2022), or National Cancer Center Hospital (2012–2015) were included, with institutional review board approval obtained at each site (approval numbers: M09-0551, M20-0007, G0683, 2022083Ge, 2022-003). Eight patients with grade 1 to 2 endometrioid carcinomas were also included. Tissue samples from the National Cancer Center Hospital were provided by the National Cancer Center Biobank, Japan. This study was conducted as a retrospective, noninterventional analysis; therefore, prospective trial registration and a registry identifier were not applicable. All pathologists involved in histopathologic diagnosis, as well as analysts performing genomic and transcriptomic analyses, were blinded to clinical outcomes and prognostic information.

### Whole-exome sequencing

Tumor tissues were collected immediately after surgery, cut into small pieces, and stored at −80°C until use. Genomic DNA was extracted from surgically resected specimens using the QIAamp Fast DNA Tissue Kit (Qiagen). Peripheral blood samples were stored at −30°C. Genomic DNA was extracted from peripheral blood samples using the QIAamp DNA Blood Midi Kit (Qiagen). Whole-exome sequencing (WES) libraries were generated using the Twist Library Preparation EF system (Twist Bioscience) with enzymatic fragmentation, the Twist Universal Adaptor system, and the Twist Fast Hybridization Target Enrichment system. Briefly, 50 ng of genomic DNA was enzymatically fragmented to 200 to 300 bp, followed by end-repair, A-tailing, and pair-end index adapter ligation. Precapture amplification was conducted with KAPA HiFi DNA polymerase (KAPA Biosystems, Roche Diagnostics). Exonic fragments from approximately 750 ng of amplified products were enriched using the Twist Comprehensive Exome Panel as a probe. Massively parallel, paired-end sequencing of sample libraries was performed with a NovaSeq6000 sequencer (Illumina, RRID: SCR_016387).

### Analysis of sequence data

Reads from paired-end WES were aligned to the human reference genome (hg38) using the Burrows–Wheeler Aligner (http://bio-bwa.sourceforge.net/, RRID: SCR_010910) and Bowtie 2 (version 2.1.0, http://bowtie-bio.sourceforge.net/bowtie2/index.shtml, RRID: SCR_016368). Somatic (synonymous and nonsynonymous) mutations were called using an in-house caller and publicly available mutation callers: Genome Analysis Toolkit (https://gatk.broadinstitute.org/hc/en-us, RRID: SCR_001876), MuTect2 (version 2.7-2, https://gatk.broadinstitute.org/hc/en-us/articles/360037593851-Mutect2, RRID: SCR_026692), and VarScan 2 (http://varscan.sourceforge.net/). Mutations were discarded if any of the following criteria were met: total read number <20, variant allele frequency (VAF) in tumor samples <0.05, mutant read number in germline control samples >2, mutation in only one genome strand, or variant present in normal human genomes of the 1,000 Genomes Project dataset (https://www.internationalgenome.org/, RRID: SCR_006828) or the in-house database. Gene mutations were annotated using SnpEff (http://snpeff.sourceforge.net, RRID: SCR_005191). CN was analyzed using an in-house pipeline that determined the log R ratio (LRR) as follows: (i) homozygous (VAF ≤0.05 or ≥0.95) or heterozygous (VAF 0.4–0.6) single-nucleotide polymorphisms (SNP) were selected from the genomes of the related normal samples in the 1,000 Genomes Project database; (ii) normal and tumor read depths for the selected SNPs were adjusted according to the G + C percentage of a 100-bp window flanking position; (iii) LRR was calculated as log_2_(*t*_*i*_/*n*_*i*_), in which *n*_*i*_ and *t*_*i*_ are normal and tumor-adjusted depths at position *i*, respectively; and (IV) each representative LRR was determined using the median of a moving window (1 Mb) centered at position *i*. The LRR of the CN for both alleles, i.e., the major and minor alleles, was determined for every region of the genome. Allele-specific CN was inferred using the LRR in FACETS (version 0.6.2, RRID: SCR_026264; ref. 28). The variant-calling strategy and key parameters used in this study are summarized in Supplementary Table S2.

### Analysis of mutational signatures

Mutations with VAF ≥0.1 that passed the quality filter were subjected to mutational signature analysis with SigProfilerExtractor (https://github.com/AlexandrovLab/SigProfilerExtractor, version 1.1.21, RRID: SCR_023121; ref. 29). To summarize results, we aggregated single-base substitution (SBS) categories into broader categories. The age category was derived by summing the values from SBS1 (age) and SBS5 (age). The APOBEC category combined values from SBS2 (APOBEC) and SBS13 (APOBEC). Similarly, the POLE category was created by summing values from SBS10a (POLE), SBS10b (POLE), SBS14 (POLE), and SBS20 (POLD1). The MMRd category was based on values in SBS15 (MMRd). SBS87 was used as is. According to the description provided on the Catalogue Of Somatic Mutations in Cancer website (https://cancer.sanger.ac.uk/signatures/, RRID: SCR_002260), we also aggregated CN signature categories into broader categories. The HRD-TandemDup category was derived by summing the values from CN17 [homologous recombination deficiency (HRD)], CN18 (unknown), and CN19 (unknown).

### Transcriptome sequencing

Ribosomal RNA depletion using NEBNext rRNA Depletion Kit v2 (New England Biolabs) was performed on 500 ng of RNA extracted from clinical samples. Sequencing libraries were prepared using the NEBNext Ultra II RNA Library Prep Kit for Illumina (New England Biolabs) and sequenced over 150 bp from both ends using the NovaSeq6000 sequencer (Illumina). The expression level of each gene was calculated using DESeq2 (https://bioconductor.org/packages/release/bioc/html/DESeq2.html, version 1.48.1, RRID: SCR_015687).

### Calculation of phenotypic score

Phenotypic scores representing glandular/luminal, ciliated, and EMT-like transcriptional programs were calculated using single-sample gene set enrichment analysis (ssGSEA). Gene sets were derived from differentially expressed genes between transcriptomic clusters and further refined by manual curation to select representative genes with strong expression contrast and biological relevance. A rank-based ssGSEA approach was chosen to enhance robustness across samples and datasets. We determined ssGSEA scores using the GSEAPY package (https://gseapy.readthedocs.io/en/latest/, version 1.0.6, RRID: SCR_025803; ref. 30).

### MSI testing

Using labeled primers, MSI was analyzed with polymerase chain reaction (PCR) at five microsatellite loci: BAT25, BAT26, NR-21, NR-24, and MONO-27. PCR products were electrophoresed on a 3730xl DNA Analyzer (Applied Biosystems, Thermo Fisher Scientific, RRID: SCR_018059) and analyzed using Peak Scanner 2 software (Applied Biosystems).

### TCGA data analysis

We obtained batch effect–normalized RNA sequencing (RNA-seq) data for uterine corpus endometrial carcinoma and uterine carcinosarcoma from TCGA pan-cancer dataset available at UCSC Xena (RRID: SCR_018938; ref. 31). Metadata, such as histologic type and subtype information, were acquired from both UCSC Xena and cBioPortal (https://www.cbioportal.org/, RRID: SCR_014555). The analysis was conducted using 595 samples (including adjacent normal tissues) and 17,507 genes (with no missing values) for which gene expression data and metadata were available.

### IHC analysis and interpretation

All IHC tests, including those for p53 (DO7, prediluted, Dako, Glostrup, Denmark, RRID: AB_3669092), PMS2 (EP51, prediluted, Dako/Agilent, RRID: AB_2889977), MSH6 (EP49, prediluted, Dako/Agilent, RRID: AB_2889975), estrogen receptor (ER; SP1, prediluted, Ventana/Roche, RRID: AB_2335977), progesterone receptor (PR; 1E2, prediluted, Ventana/Roche, RRID: AB_2335976), CD8 (4B11, 1:200, Leica/Novocastra, RRID: AB_442068), and PD-L1 (E1L3N, 1:400, Cell Signaling Technology, RRID: AB_2687655) were performed as described in our previous study. IHC testing for p53, PMS2, MSH6, CD8, and PD-L1 was performed with the Link 48 autostainer (Dako/Agilent, RRID: SCR_026889), whereas ER and PR were stained using the BenchMark ULTRA platform (Ventana/Roche, RRID: SCR_025506). After deparaffinization, tissue sections were stained using the antibodies mentioned above and then counterstained with hematoxylin. For PD-L1 evaluation, the E1L3N clone was used, which has been shown to be highly concordant with clinically validated assays (32, 33), and internal validation confirmed concordance using combined positive score (CPS) ≥1.

Pathologists evaluated all IHC slides according to the following criteria. For p53 staining, tumors with either a strong and diffuse nuclear staining pattern (>80% of carcinoma cells) or a staining pattern entirely negative of carcinoma cells (null pattern) were considered to have aberrant p53 staining pattern indicating *TP53* alterations. Staining of the adjacent nontumor cells served as an internal positive control. Tumors with weak or heterogeneous staining patterns were considered to have WT *TP53*. In addition, subclonal mutant p53 immunostaining was defined as a combination of WT patterns and one or more mutant patterns, with each present in at least 5% of tumor cells (34). Because IHC for PMS2 and MSH6 can reportedly be used instead of the four-antibody panel (MLH1, MSH2, MSH6, and PMS2) for MMRd screening (35), MMRd status was defined as the complete loss of nuclear staining for PMS2 or MSH6 proteins in this study. Internal positive controls with intact nuclear staining included the adjacent normal mucosa, stromal cells, and inflammatory cells. For prognostic analyses, ER was dichotomized using a 10% cutoff (negative, <10% vs. positive, ≥10%) per the ESGO/ESTRO/ESP guidelines. CD8^+^ T cells were analyzed using HALO software (Indica Lab, RRID: SCR_018350). Regions of interest were manually annotated by a board-certified pathologist (S. Sakashita), including viable tumor areas and excluding necrosis, hemorrhage, artifacts, and tissue folds. Cell segmentation was performed using HALO Multiplex IHC v3.4.9 module with hematoxylin-based nuclear detection and watershed separation. Positive thresholds were defined using OD histogram inspection to match visual interpretation. Thresholds were fixed and applied uniformly across all slides. All results were visually reviewed to confirm appropriate segmentation and thresholding. The number of CD8^+^ T cells and their area within that region were measured, and the number of CD8^+^ T cells per unit area was calculated. PD-L1 expression was assessed by calculating the CPS (36). The CPS was calculated by dividing the number of PD-L1–positive cells (including viable tumor cells, lymphocytes, and macrophages) by the total number of viable tumor cells and multiplying by 100. Representative IHC staining results are shown in Supplementary Fig. S3A–S3J. All IHC scoring in this study was performed by a single board-certified pathologist with subspecialty expertise in gynecologic pathology (H. Yoshida). IHC was performed on formalin-fixed, paraffin-embedded (FFPE) diagnostic specimens using a single automated staining run per case. Technical replicates were not generated, as assay robustness was ensured through validated preanalytic procedures and internal and external controls, consistent with standard clinical pathology practice.

### Definition of molecular subtypes (i) genomic and (ii) pathologic

(i) Genomic: Tumors were classified into four molecular subtypes as follows. The *POLE*-ultramutated subtype was defined by the presence of pathogenic mutations located at the established hotspot positions within the exonuclease domain of the *POLE* gene (such as P286R, S297Y, V411L, L424I, and S459F; https://www.oncokb.org/), which are known to impair exonuclease activity (37). The MSI-H subtype was assigned to tumors with high MSI status and/or high MSI score, in the absence of *POLE* hotspot mutations. The *TP53*-mutated (*TP53*-mut) subtype included tumors with pathogenic *TP53* mutations (or *MDM2* amplification as a negative regulator of *TP53*), after excluding POLE and MSI-H cases. Tumors that did not meet criteria for any of the above three subtypes were classified as NSMP. As we compared our data with the TCGA dataset, we primarily relied on WES data for molecular classification, whereas IHC was also performed in our cohort.
(ii) Pathologic (IHC-based): The *POLE*-ultramutated subtype was defined in the same manner as in (i). MMRd was defined as the loss of either PMS2 or MSH6 expression by IHC (35). The p53-abn subtype was defined by abnormal p53 IHC staining patterns. Tumors not meeting criteria for these categories were classified as NSMP.

### Statistical analyses

Statistical analyses were performed using Python (version 3.11.4, RRID: SCR_008394) and SciPy (version 1.11.1, RRID: SCR_008058). Quantitative variables were compared between groups using either the nonparametric Wilcoxon rank-sum test or one-way analysis of variance (ANOVA), followed by Tukey *post hoc* test where applicable, and a two-sided *P* value < 0.05 was considered statistically significant for these comparisons. For gene expression–based analyses at the gene set level, including GSEA and ssGSEA, multiple-comparison correction was performed using false discovery rate control as implemented in the respective analysis packages. Statistical analyses were exploratory in nature, and exact *P* values are reported; a nominal *P* value < 0.05 was used as a reference threshold for interpretation. Correlations between variables were evaluated using Spearman correlation coefficients. Because this study was conducted as a retrospective observational analysis, randomization and prospective group allocation were not applicable.

## Results

### Classification of high-grade endometrial carcinomas based on the results of exome sequencing

**Figure 1.**
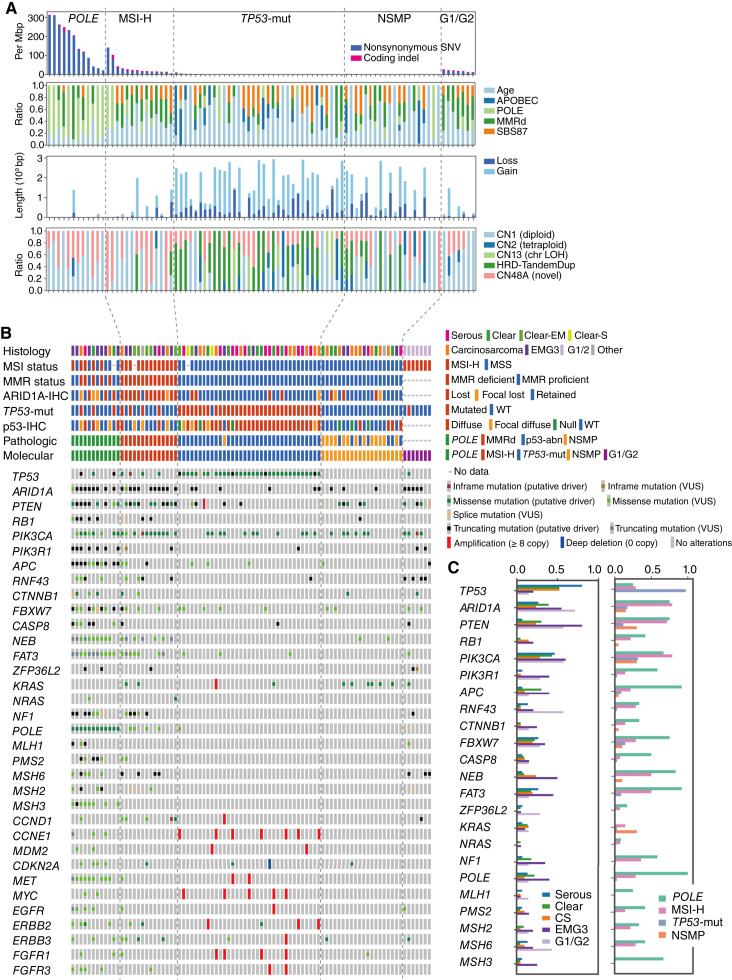
Exome sequencing revealed genomic landscape across high-grade endometrial cancers. **A,** Bar plots showing the number of mutations and signature proportions. The top shows the number of mutations per megabase. The middle shows the relative contribution of different mutational signatures. The bottom two show the length of genomic regions with CN loss or gain and the relative contribution of different CN signatures. **B,** Summary of genetic and clinical features for each sample. Each column corresponds to a sample. Each row corresponds to a different genetic or clinical feature or gene mutation status. **C,** Bar plots showing the frequency of specific gene mutations by subtype. The left shows the frequency of mutations in various genes by histologic classification. The right shows mutation frequency by molecular subtype. APOBEC, apolipoprotein B mRNA-editing enzyme, catalytic polypeptide; clear-S, clear cell carcinoma mixed with serous carcinoma; CS, carcinosarcoma; EM, endometrioid; G1/G2, grades 1–2 endometrioid; MSS, microsatellite stable; VUS, variant of unknown significance.

We conducted exome sequencing (tumor–normal paired) to detect genomic mutations in 81 tumors, encompassing nonendometrioid tumors as well as EMG3s (Fig. 1A–C; Supplementary Fig. S4; Supplementary Description; Supplementary Table S3). Clinicopathologic features are summarized in Supplementary Table S4. The overall mutation profile aligned well with previous studies (2, 10, 38, 39). However, we also observed differences in mutation frequencies compared with the TCGA dataset, notably a higher frequency of *POLE* mutations within exonuclease domain and a lower frequency of *CTNNB1* mutations (Fig. 1B). These discrepancies may reflect differences in pathologic characteristics, namely, exclusive inclusion of high-grade endometrial carcinomas. They may also be influenced by potential racial or ethnic backgrounds. Additionally, we performed mutational signature, chromosomal CN, and CN signature analyses (Fig. 1A).

### Molecular subtype classification based on (i) genomic data and (ii) IHC data

**Figure 2.**
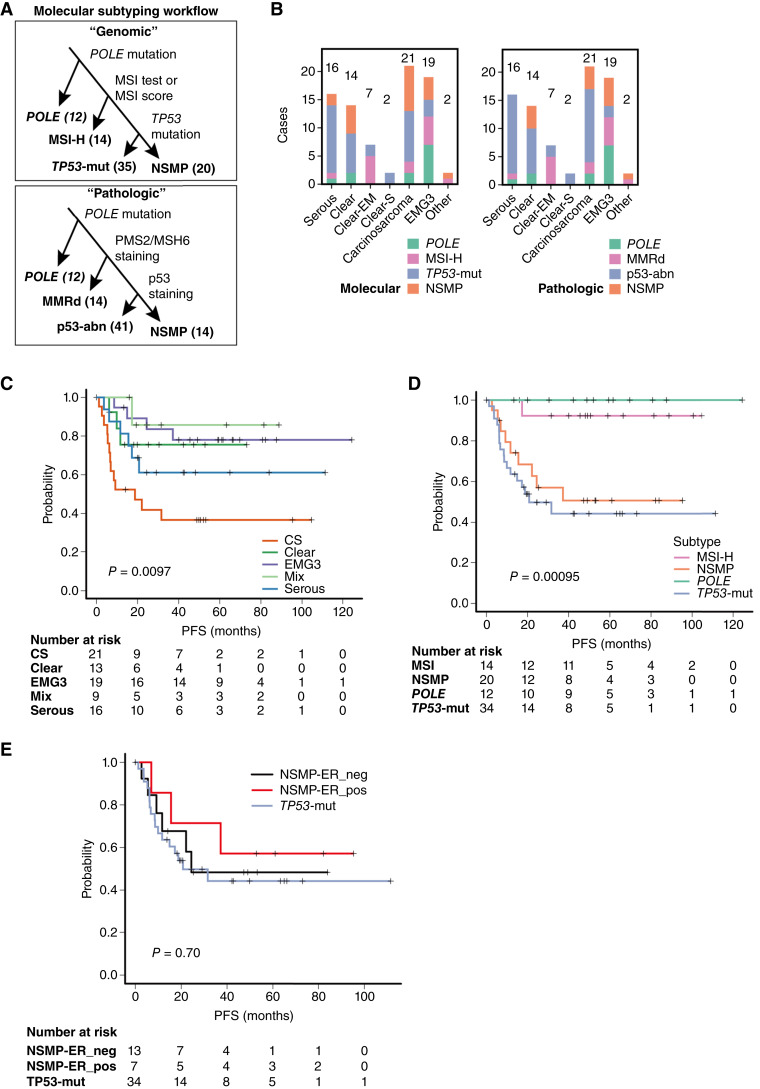
Relationship between histologic classification and molecular subtype. **A,** Workflow for determination of molecular subtypes. **B,** Bar plots showing the distribution of molecular subtypes across different histologic classifications. The numbers above the bars indicate the number of patients in each category. **C,** Kaplan–Meier curves for PFS according to histologic subtype. **D,** Kaplan–Meier curves for PFS according to molecular subtype. **E,** Kaplan–Meier curves for PFS according to ER expression. *P* values were calculated using the log-rank test. Numbers at risk are shown below each graph. EM, endometrioid.

Tumors were classified into four molecular subtypes, including *POLE*, MSI-H, *TP53*-mut, and NSMP, based on genomic data and validated with IHC findings, as outlined in Fig. 2A. This approach was chosen to ensure both clinical relevance and consistency with TCGA-derived frameworks, while addressing the limited sensitivity of CN-based classification in samples with low tumor content. Genomically (i), 12 (14.8%) were classified as *POLE*, 14 (17.3%) as MSI-H, 35 (43.2%) as *TP53*-mut, including 34 with *TP53* mutations and 1 with *MDM2* amplification, and 20 (24.7%) as NSMP (Fig. 2A). Pathologically (ii), 12 (14.8%) were classified as *POLE*, 14 (17.3%) as MMRd, 41 (50.6%) as p53-abn, and 14 (17.3%) as NSMP.

Among non-*POLE*/non–MSI-H cases, discordance between *TP53* mutation status and p53 IHC was observed in 8 of 55 cases (14.5%). In addition, discrepancies between CN-based classification and *TP53*/p53 status were identified in 16 of 55 cases (29.1%; Supplementary Table S5; Fig. 2A). This discordance seems to be a characteristic feature of high-grade endometrial carcinomas, in which chromosomal instability and complex allelic configurations are frequent. Detailed case-level analyses (Supplementary Tables S5–S8; Supplementary Description) suggest that this discrepancy is consistent with technical and biological factors, including (i) *TP53* alterations missed by WES (e.g., large deletions, other structural alterations, or variants excluded by strict filtering), (ii) spatial heterogeneity arising from distinct sampling for sequencing (fresh-frozen) versus immunostaining (FFPE), and (iii) chromosomal instability arising from mechanisms independent of *TP53* deficiency or falling below predefined CN-H thresholds (Supplementary Descriptions). These findings underscore the need for improved stratification strategies for non-*POLE*/non–MSI-H high-grade endometrial carcinomas.

In the following analyses, *TP53* mutation status was used as an operational stratification axis to examine the relationship among histologic, phenotypic, and genotypic features of high-grade endometrial carcinoma.

### Correlation between histologic type and genomic abnormalities in high-grade endometrial carcinoma

We examined the distribution of molecular subtypes across histologic types in high-grade endometrial carcinoma (Fig. 2B). As expected, *TP53* mutations were frequent in serous carcinomas (14/18, 78%), whereas MSI-H/MMRd and *POLE* subtypes were the most predominant in endometrioid carcinomas, including those with mixed histology (e.g., mixed with clear cell components). Clear cell carcinomas and carcinosarcomas were distributed across multiple molecular subtypes, particularly *TP53*-mut and NSMP (Fig. 2B).

As shown, histologic classification and molecular subtypes do not always align in high-grade endometrial carcinoma. This discrepancy raises several important clinical questions, such as what are the clinical and biological differences between *TP53*-mut carcinosarcomas and serous carcinomas and which factors determine their divergent histologic phenotypes despite sharing the common genotypes.

### Prognostic stratification by histologic, molecular classification, and CN-based classification

Survival analysis revealed that whereas histologic subtypes showed prognostic differences, particularly poor outcomes in carcinosarcoma, molecular classification provided clearer stratification (Fig. 2C and D). *POLE* and MSI-H tumors showed favorable prognoses, whereas NSMP type exhibited similarly poor prognosis to *TP53*-mut type. Recent studies have reported that prognosis within NSMP varies according to ER status (27, 40). We performed survival analysis stratifying NSMP tumors into ER-positive and ER-negative groups. In this high-grade–restricted cohort, no significant prognostic difference was observed between the two groups (*P* = 0.70, Fig. 2E), and even ER-positive NSMP cases exhibited poor outcomes comparable with *TP53*-mut tumors. This suggests that although ER stratification is highly effective in broader cohorts to distinguish low-risk (typically low-grade) from high-risk tumors, its prognostic utility may be attenuated in the high-grade setting in which histologic aggressiveness predominates.

**Figure 3.**
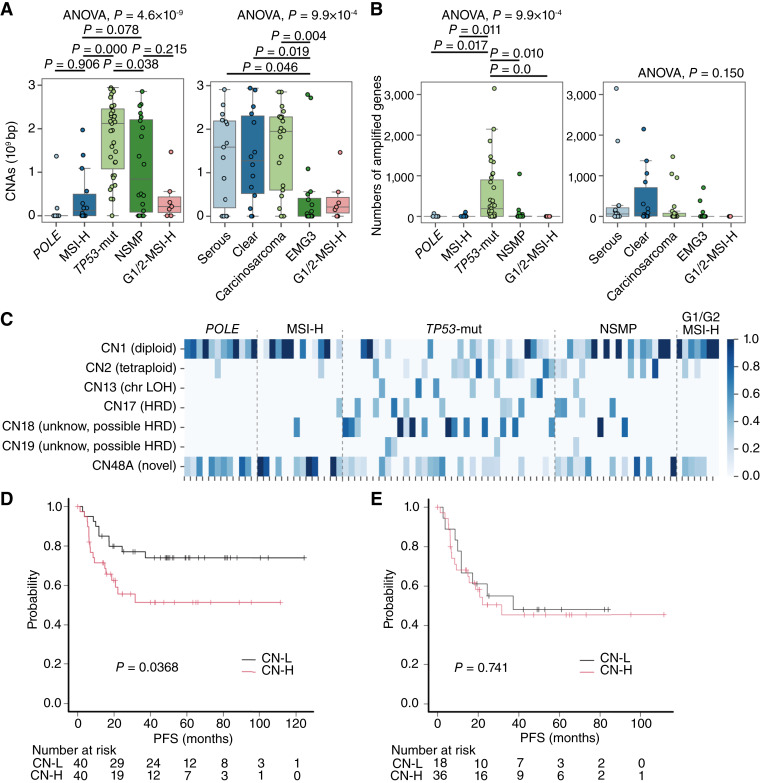
Differences in chromosome CN status by molecular subtype. **A,** Box plots showing the length of genomic regions with CNAs across different molecular subtypes or histologic subtypes. Statistical comparisons were performed using ANOVA followed by Tukey *post hoc* analysis. **B,** Box plots showing the number of amplified genes across different molecular subtypes or histologic subtypes. ANOVA *P* values are provided for each comparison. **C,** Heatmap showing the CN signatures of various chromosomal regions across different molecular subtypes. **D,** Kaplan–Meier curves for PFS according to CN status. **E,** Kaplan–Meier curves for PFS according to CN status for *TP53*-mut and NSMP patients. *P* values were calculated using the log-rank test. ANOVA, analysis of variance; APOBEC, apolipoprotein B mRNA-editing enzyme, catalytic polypeptide; G1/G2, grades 1–2 endometrioid; LOH, loss of heterozygosity.

Given the similarly poor outcomes in both *TP53*-mut type and NSMP type, we investigated copy-number alterations (CNA) to further distinguish these subtypes. *TP53*-mut type showed more extensive CNAs than NSMP type (with the median length of CNA regions measuring 2.12 Gbp in *TP53*-mut type and 844 Mbp in *NSMP* type), with frequent amplifications in genes such as *CCNE1*, *ERBB2*, and *FGFR1* and a higher prevalence of the HRD-associated CNA signature CN18 (Figs. 1B and 3A–C). In contrast, the BRCA1/2 deficiency–associated CNA signature CN17 was rare, and the corresponding mutational signature SBS3 was absent; instead, age, APOBEC, or SBS87 signatures were predominant in the *TP53*-mut and NSMP subtypes (Figs. 1A and 3C). Although CNA burden was associated with poorer prognosis in the entire cohort (Fig. 3D), this effect was largely driven by inclusion of *POLE* and MSI-H types with minimal CNAs. Within *TP53*-mut and NSMP types, CNA-based classification (CN-H vs. CN-L) failed to stratify prognosis (Fig. 3E), indicating its limited utility in high-grade endometrial carcinoma. These findings highlight the limited prognostic resolution of current molecular classifiers in high-grade endometrial carcinoma, particularly for NSMP, and the necessity of further prognostic stratification with a deeper understanding of the biological characteristics.

### Clustering of tumors based on the global gene expression profile

**Figure 4.**
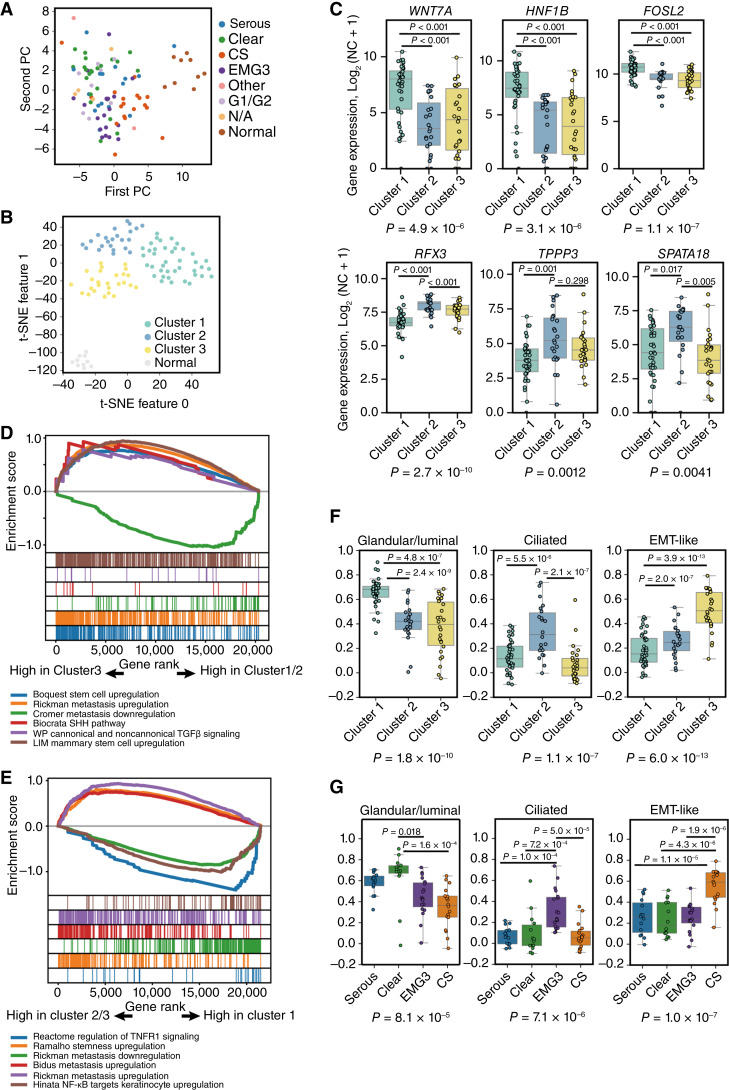
Clustering of high-grade endometrial carcinomas based on gene expression profiles. **A,** Principal component analysis plot of transcriptome expression showing the distribution of samples in association with their histologic type. **B,** The t-SNE plot shows the results of hierarchical clustering, indicating RNA cluster classifications. **C,** Box plots of gene expression levels for selected genes across RNA clusters. The *y*-axis represents the log_2_(normalized count + 1) of gene expression. *P* values are provided for comparisons based on ANOVA followed by Tukey *post hoc* analysis. **D** and **E,** GSEA plots showing representative pathways with nominal enrichment in different RNA clusters. Corresponding nominal *P* values and FDR q-values are listed in Supplementary Table S9. **D,** Enrichment scores for cluster 3 compared with clusters 1 and 2. **E,** Enrichment scores for cluster 1 compared with clusters 2 and 3. **F,** Box plots showing the distribution of phenotypic scores across RNA clusters. **G,** Box plots showing the distribution of phenotypic scores across histologic subtypes. ANOVA, analysis of variance; CS, carcinosarcoma; EM, endometrioid; G1/G2, grades 1–2 endometrioid; N/A, not available; NC, normalized count; PC, principal component; t-SNE, *t*-distributed stochastic neighbor embedding.

To identify clinically relevant features beyond molecular and histopathologic classifications in high-grade endometrial carcinoma, we performed transcriptomic profiling. We selected 5,000 genes with high expression levels and significant variability across samples for clustering. Principal component analysis revealed loose grouping patterns for EMG3s, carcinosarcomas, and clear cell carcinomas, whereas serous carcinomas showed more dispersed patterns (Fig. 4A). To enhance cluster resolution, we used *t*-distributed stochastic neighbor embedding, which revealed one distinct cluster of normal tissues and three transcriptionally defined tumor clusters (Fig. 4B).

We further characterized the cellular identity of these clusters. Cluster 1 exhibited elevated expression of genes associated with glandular/luminal endometrial cells, such as *WNT7A*, *HNF1B*, and *FOSL2* (Fig. 4C). Cluster 2 was enriched for ciliated cell markers, including expression of *RFX3* and *TPPP3*, along with *ESR1* (encoding ER) and *PGR* (encoding PR) (Fig. 4C). Cluster 3 showed high expression of Hedgehog pathway genes (*GLI1* and *GLI2*), prolactin (*PRL*), and mesenchymal markers (*TWIST1*, *ZEB1*, and *CDH2*), consistent with the carcinosarcoma phenotype (Supplementary Fig. S5).

To explore the biological relevance of these clusters, we performed GSEA. Cluster 3 showed nominal enrichment of basal-like or stem-like signatures, along with elevated TGFβ signaling, consistent with EMT and a sarcomatous phenotype (Fig. 4D; Supplementary Table S9). In contrast, cluster 1 showed nominal enrichment of TNF and NF-κB pathway activity (Fig. 4E; Supplementary Table S9), indicative of a proinflammatory state and enhanced immune response with potential responsiveness to ICIs.

To generalize these phenotypes, we defined gene signatures representing glandular/luminal, ciliated, and EMT-like features and calculated ssGSEA scores (Supplementary Table S10). These phenotypic scores aligned well with the transcriptomic clusters and also correlated with histologic classifications (Fig. 4F and G). Clear cell carcinomas predominantly exhibited strong glandular/luminal features, whereas EMG3s were enriched for ciliated features.

### Transcriptomic phenotypes reconnect histologic features and refine the molecular classification

**Figure 5.**
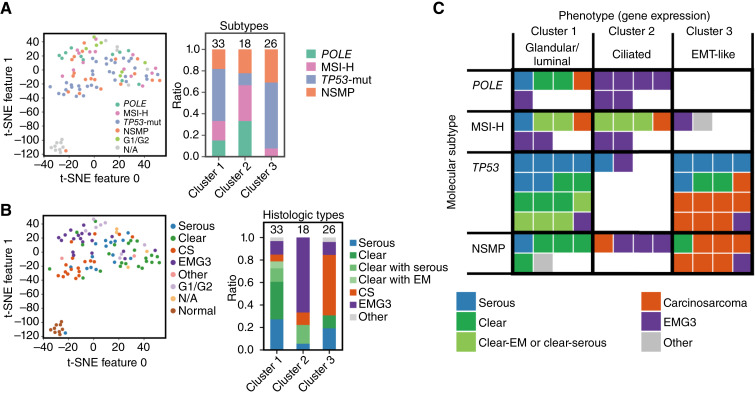
Integration of transcriptomic phenotypes with molecular and histologic classifications in high-grade endometrial carcinoma. **A,** The t-SNE plot shows the distribution of samples across different molecular subtypes. **B,** The t-SNE plot shows the distribution of samples across different histologic types. **C,** The relationship between molecular subtypes and phenotypes (RNA clusters) is shown in association with histologic subtypes (distinguished by color). Each colored block represents a specific histologic subtype: blue, serous carcinomas; green, clear cell carcinomas; light green, clear endometrioid or clear serous; red, carcinosarcoma; purple, EMG3; and gray, other. t-SNE, *t*-distributed stochastic neighbor embedding.

We next examined the clinical and immunologic significance of the identified transcriptomic phenotypes. The three clusters showed partially concordant distributions with both molecular and histologic subtypes. Cluster 2 predominantly contained *POLE* and MSI-H tumors, whereas cluster 3 was enriched for *TP53*-mut and NSMP tumors (Fig. 5A). NSMP tumors were scattered across all three clusters, indicating heterogeneity within this molecular group.

In terms of histology, EMG3 tumors were mainly found in cluster 2, clear cell carcinomas in cluster 1, and carcinosarcomas in cluster 3 (Fig. 5B). Although serous carcinomas are often treated as a single high-grade entity with *TP53* alterations, they were distributed across clusters, particularly clusters 1 and 3, suggesting underlying transcriptional heterogeneity within this histologic group (Fig. 5B). The clustering pattern was largely reproducible in the TCGA dataset, aside from the absence of clear cell carcinomas (Supplementary Figs. S6A–S6E and S7; Supplementary Description; Supplementary Table S11).

A summary matrix further illustrated the correspondence between transcriptomic phenotypes and molecular/histologic subtypes (Fig. 5C). Carcinosarcomas matched the EMT-like (stem-like) phenotype in cluster 3, whereas *TP53*-mut type and *POLE* type showed phenotypic heterogeneity, appearing across multiple clusters with differing histologic features. These results highlight the added resolution transcriptome-based classification provides beyond molecular subtyping alone and support its potential clinical relevance.

### Transcriptomic phenotype–based classification reveals distinct immunologic and clinical features

**Figure 6.**
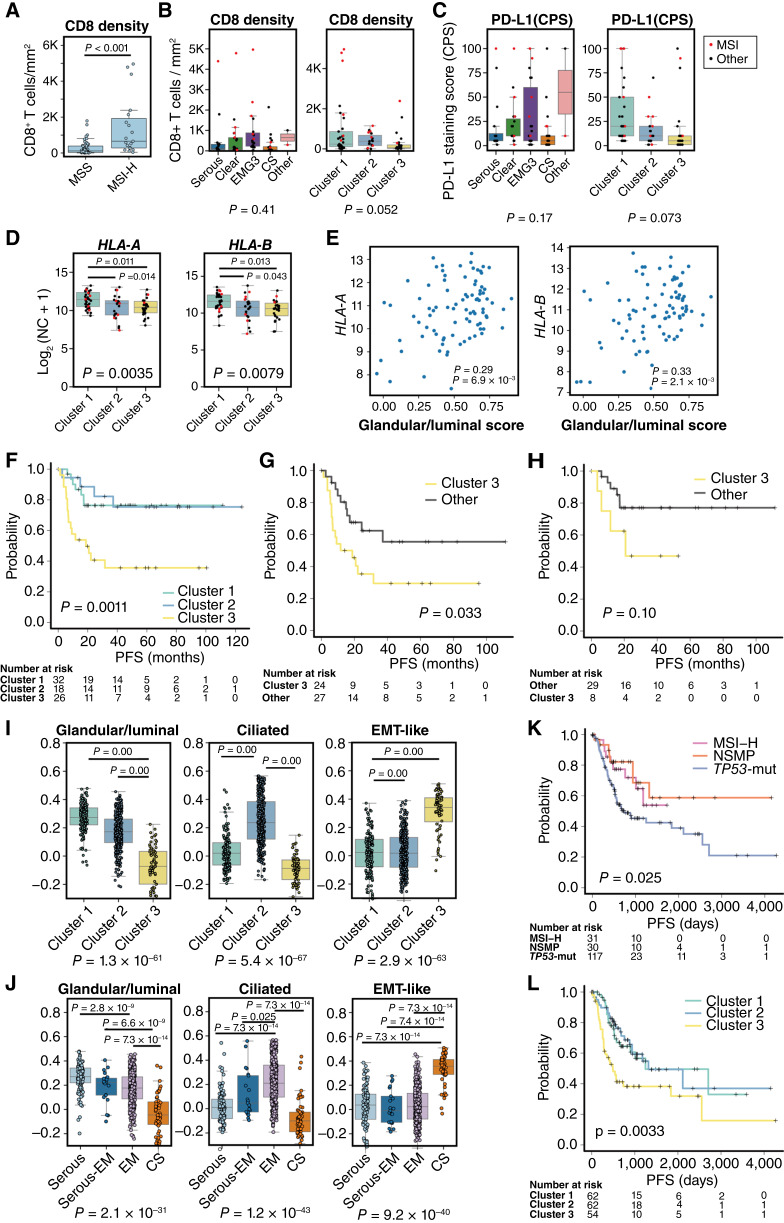
Differential molecular, immunologic, and clinical features among transcriptomic clusters in high-grade endometrial carcinoma. **A,** Box plot showing the density of CD8^+^ T cells in MSS and MSI-H samples. **B,** Box plots showing the density of CD8^+^ T cells across different histologic subtypes and RNA clusters. **C,** Box plots showing the PD-L1 staining across different histologic subtypes and RNA clusters. **D,** Box plots showing the expression of *HLA-A* and *HLA-B* across different RNA clusters. **E,** Scatter plots showing the correlation between glandular/luminal score and the expression of *HLA-A* and *HLA-B*. Spearman rank correlation coefficients and *P* values are indicated. **F,** Kaplan–Meier curves for PFS according to RNA clusters. **G,** Kaplan–Meier curves for PFS according to RNA clusters for *TP53*-mut patients and NSMP patients. *P* values were calculated using the log-rank test. **H,** Kaplan–Meier curves for PFS according to RNA clusters for patients with serous and clear cell carcinoma. *P* values were calculated using the log-rank test. **I,** Box plots showing the distribution of phenotypic scores across RNA clusters in the TCGA dataset. **J,** Box plots showing the distribution of phenotypic scores across histologic subtypes in the TCGA dataset. **K,** Kaplan–Meier curves for PFS of MSI-H, *TP53*-mut, and NSML patients in the TCGA dataset. **L,** Kaplan–Meier curves for PFS of the same patient groups in the TCGA dataset, stratified by RNA clusters. NC, normalized count.

Interestingly, MSI-H tumors were distributed across all transcriptomic clusters (Fig. 5A), suggesting that MSI status alone may not fully capture the heterogeneity in immune response or ICI sensitivity. To further explore immunologic characteristics across clusters, we assessed immunologic phenotypes, including CD8^+^ T-cell infiltration and PD-L1 expression. As expected, MSI-H tumors exhibited higher CD8^+^ T-cell infiltration compared with MSS tumors (Fig. 6A). However, CD8^+^ infiltration did not significantly differ between cluster 1 and clusters 2/3 and likewise showed no significant differences among histologic subtypes overall (Fig. 6B). PD-L1 expression patterns observed by IHC closely linked with CD8^+^ infiltration, driven partly by CD8^+^ enrichment in a subset of cluster 1 tumors, regardless of MSI status (Fig. 6C). This may reflect enhanced antigen presentation, as *HLA-A* and *HLA-B* expression levels were significantly higher in cluster 1 compared with clusters 2 and 3 (Fig. 6D), and both genes positively correlated with glandular/luminal scores (Fig. 6E), suggesting a link between epithelial phenotype and immune activation. We further repeated these analyses after excluding *POLE*/MSI-H types and confirmed that *HLA-A* and *HLA-B* expression remained higher in cluster 1 than in clusters 2 and 3 and maintained a positive correlation with the glandular/luminal score, indicating that the association between cluster 1 and immune activation is not driven by *POLE*/MSI-H status (Supplementary Fig. S8A–S8H). These trends were corroborated in the TCGA dataset (Supplementary Fig. S9A and S9B). Collectively, MSI status remains a primary driver of T-cell infiltration, but cluster 1 status serves as a primary marker of PD-L1 expression, as well as *HLA-A* and *HLA-B*.

Finally, we examined PFS across clusters. Patients in cluster 3, which is characterized by EMT-like transcriptomic features and enriched for carcinosarcoma, had significantly shorter PFS compared with clusters 1 and 2 (Fig. 6F). Notably, even within the NSMP and *TP53*-mut types, cluster 3 was associated with significantly poorer PFS (Fig. 6G), suggesting that transcriptomic phenotyping may provide additional context alongside genome-based molecular subtyping. The poorer PFS was also observed when limited to the *TP53*-mut subtype (Supplementary Fig. S10).

However, when carcinosarcoma cases were excluded, no statistically significant difference in PFS was observed among clusters, indicating that the prognostic impact of cluster 3 is largely influenced by histologic composition. An exploratory analysis restricted to serous and clear cell carcinomas showed a tendency toward poorer PFS in cluster 3, although this did not reach statistical significance due to the limited sample size (Fig. 6H).

Transcriptomic clustering was recapitulated in the TCGA dataset (Supplementary Figs. S6A–S6E and S7), with ssGSEA scoring confirming concordant assignment of cases to the three transcriptomic phenotypes (Fig. 6I and J). PFS analysis, excluding grade 1/2 endometrioid carcinomas, exhibited the separation between NSMP and *TP53*-mut tumors that is in line with the general concept but in contrast to our cohort (Fig. 6K). Still, it showed the poorest outcomes for cluster 3 (Fig. 6L), supporting the reproducibility of transcriptomic phenotyping and its association with clinical outcome patterns. However, evaluation of the prognostic relevance of cluster 3 independent of histology was not feasible due to the small number of noncarcinosarcoma cases assigned to this cluster in TCGA. Collectively, these findings suggest that EMT-like transcriptional features captured by cluster 3, although closely linked to carcinosarcoma, can complement genome-based molecular subtypes by capturing transcriptional programs associated with aggressive clinical behavior.

## Discussion

In this study, we integrated genomic, histopathologic, and IHC analyses of high-grade endometrial carcinomas, revealing classification ambiguity, particularly discordance among *TP53* mutation status, p53 IHC, and CN-based classification. Because the original TCGA framework was derived from cohorts including both low- and high-grade tumors, CN-high cases have been largely equated with p53-abn status in ProMisE; however, our high-grade–restricted analysis demonstrates that this equivalence does not consistently hold when only high-grade endometrial carcinomas are considered. In addition, by incorporating transcriptomic phenotypes alongside established molecular frameworks, our findings highlight phenotypic heterogeneity that is not fully captured by current molecular subtypes alone.

Molecular subtype analysis confirmed expected associations (2, 11, 39, 41–44) such as the predominance of *TP53*-mut tumors in serous carcinoma and the enrichment of *POLE* mutations in high-grade endometrial carcinoma. *TP53*-mut tumors were characterized by extensive chromosomal CNAs, frequent *CCNE1* amplification, and a high prevalence of the HRD-associated CNA signature CN18. In contrast, CNA signature CN17 and mutational signature SBS3, both typically linked to BRCA1/2-deficient homologous recombination repair, were largely absent, consistent with previous reports showing a low prevalence of SBS3 in endometrial carcinomas (45, 46). These findings suggest that the observed HRD-like CNA pattern in *TP53*-mut tumors likely arises through mechanisms independent of BRCA1/2 loss, unlike the canonical HRD observed in ovarian and breast cancers (47, 48). The high frequency of *CCNE1* amplification and low frequency of *BRCA1*/*2* mutations further support a high prevalence of HR proficiency in high-grade endometrial carcinomas (49, 50). Notably, in our cohort, NSMP tumors, commonly regarded as intermediate-risk, exhibited poor clinical outcomes in the high-grade setting, comparable with *TP53*-mut tumors. Furthermore, CN-H status failed to stratify prognosis across the combined *TP53*-mut and NSMP groups, both showing similarly poor outcomes in high-grade endometrial carcinoma, highlighting the unmet need for novel stratification layers.

Transcriptomic analysis identified three phenotypes with distinct biological features. Cluster 1, an immune-responsive glandular/luminal phenotype, showed elevated HLA expression and activation of TNF/NF-κB signaling, reflecting preserved antigen-presenting capability reminiscent of well-differentiated glandular epithelium. Cluster 2, a ciliated phenotype, exhibited ER/PR activity, ciliated cell markers, and lower inflammatory activation, paralleling terminally differentiated ciliated epithelial populations. Cluster 3, an EMT-like phenotype, demonstrated high EMT and stemness signatures, low immune activity, and features of immunosuppression, consistent with aggressive biology and potential resistance to ICIs. These phenotypes align with endometrial single-cell differentiation trajectories, giving a biologically based framework for understanding tumor heterogeneity (24, 25, 51, 52).

Whereas MSI-H/dMMR status currently serves as a major biomarker for ICI eligibility, a subset of such tumors fails to respond clinically. Transcriptomic analyses in gastrointestinal tumors have revealed that nonresponders often have upregulated EMT signaling (53), suggesting that cluster 3 phenotypes may underlie ICI resistance even within MSI-H/dMMR endometrial cancers. Conversely, higher *HLA-A* and *HLA-B* expression has been associated with antigen presentation, increased T-cell infiltration and favorable prognosis in other cancers (54–56), and more generally, elevated HLA-I expression correlates with increased TIL density and treatment response to ICIs (57). These observations are consistent with our recent findings in cervical cancer, in which high expression of HLA-I was associated with increased CD8^+^ T-cell infiltration (58). Thus, serous or clear cell tumors that fall into cluster 1 might represent a subset with relatively favorable immune responsiveness (59, 60). Our cohort was collected prior to the widespread adoption of ICI-based regimens, but future studies should evaluate the relationship between transcriptomic phenotype, ICI response, and molecular subtype to enhance treatment stratification.

In high-grade endometrial carcinoma, both cluster 1 and cluster 2 were associated with more favorable PFS compared with cluster 3. This prognostic separation persisted in high-grade endometrial carcinoma even after excluding *POLE*-mut and MSI-H tumors, suggesting that *TP53*-mut and NSMP types share similarly poor prognosis. This contrasts with prior reports, including those incorporating low-grade endometrial carcinoma, in which NSMP was often considered a favorable-risk group (2, 4, 61). Although prognostic differences were observed among clusters in this cohort, these associations were strongly influenced by histologic composition, particularly carcinosarcoma, and should therefore be interpreted with caution. Integrating transcriptomic information with molecular subtype may provide a framework for contextualizing phenotypic differences within non-*POLE*/non–MSI-H tumors, warranting further investigation in more well-defined histologic subpopulations.

Our study has several limitations. First, the absence of patients treated with ICI-based regimens limited the ability to directly assess clinical response. At present, predictive biomarkers for ICI-based regimens, such as lenvatinib–pembrolizumab or chemotherapy ± PARP inhibitor with ICI, are determined primarily by MMR status. Whether transcriptomic classification can further refine prediction of ICI responsiveness remains to be evaluated in clinical settings. Second, the cohort size and composition were optimized to capture histopathologic and molecular diversity, rather than to reflect the real-world distribution of high-grade endometrial carcinoma, and a practical method for assigning transcriptomic clusters at the individual-patient level has not yet been established. Development of simplified, clinically applicable approaches to determine both molecular and transcriptomic subtypes will be essential for translation into routine practice. Lastly, cellular origin cannot be clarified on the basis of cell marker expression alone; methylome analysis, including both tumor and normal epithelial cells of various endometrial cell types, would help clarify lineage relationships.

In conclusion, our study highlights inherent challenges in the molecular classification of high-grade endometrial carcinoma, particularly in non-*POLE*/non–MSI-H tumors, that remain unresolved by current approaches. In parallel, RNA-based clustering provides complementary biological insight by contextualizing histology-associated heterogeneity beyond existing molecular subtypes.

## Supplementary Material

Supplementary Descriptions Supplementary descriptions

Supplementary Figure S1 Supplementary Figure S1. Workflow of sample processing and analytical steps.

Supplementary Figure S2 Supplementary Figure S2. Histology of high-grade endometrial carcinomas.

Supplementary Figure S3 Supplementary Figure S3. Representative results of immunohistochemical staining.

Supplementary Figure S4 Supplementary Figure S4. Microsatellite instability score and mutational signatures for each sample across different molecular subtypes.

Supplementary Figure S5 Supplementary Figure S5. Gene expression profiles across different RNA clusters in this study cohort.

Supplementary Figure S6 Supplementary Figure S6. Clustering analysis based on gene expression in the TCGA dataset.

Supplementary Figure S7 Supplementary Figure S7. Gene expression profiles across different RNA clusters in The Cancer Genome Atlas Program dataset.

Supplementary Figure S8 Supplementary Figure S8. Immunological features of tumors according to phenotypic status.

Supplementary Figure S9 Supplementary Figure S9. Analysis of immunological status in the TCGA dataset.

Supplementary Figure S10 Supplementary Figure S10. Prognostic impact of transcriptomic clusters in TP53-mutated, high-grade endometrial carcinoma.

Supplementary Table S1 Supplementary Table S1. List of samples analyzed in this study.

Supplementary Table S2 Supplementary Table S2. Significance thresholds used for GSEA and ssGSEA analyses.

Supplementary Table S3 Supplementary Table S3. Somatic nonsynonymous mutations in BRCA1 and BRCA2.

Supplementary Table S4 Supplementary Table S4. Patients’ clinicopathological characteristics.

Supplementary Table S5 Supplementary Table S5. Detailed evaluation of discordance among TP53 mutation status, p53 immunohistochemistry, and CN-based molecular classification in non-POLE/non-MSI cases.

Supplementary Table S6 Supplementary Table S6. The association between TP53 mutation status and concordance.

Supplementary Table S7 Supplementary Table S7. The association between TP53 allelic status and concordance.

Supplementary Table S8 Supplementary Table S8. The association between p53 staining pattens and concordance.

Supplementary Table S9 Supplementary Table S9. Gene set enrichment analysis statistics for representative pathways.

Supplementary Table S10 Supplementary Table S10. Selected genes to calculate phenotypic scores.

Supplementary Table S11 Supplementary Table S11. List of selected genes used for clustering.

## Data Availability

Raw sequencing data are deposited in the Japanese Genotype-phenotype Archive hosted by the DNA Data Bank of Japan (RRID: SCR_003118; ref. 62) under accession number JGAS000753/JGAD000894 (https://humandbs.dbcls.jp/en/hum0422-v1). Other data generated in this study are available upon request to the corresponding author.
