# Supplementary material for: Genomic and Transcriptomic Analysis of High-Grade Endometrial Carcinoma Reveals Biological Heterogeneity and Molecular Classification Challenges
Source: Cancer Res Commun. 2026 Apr 28;6(4):961–75. doi: 10.1158/2767-9764.CRC-25-0589 (PMC13123251; doi:10.1158/2767-9764.CRC-25-0589)
Supplement: Supplementary Descriptions [file crc-25-0589_supplementary_descriptions_suppsd.docx]

**Supplementary Descriptions**

**Genomic and transcriptomic analysis of high-grade endometrial carcinoma reveals biological heterogeneity and molecular classification challenges**

**Running title: Molecular classification challenges in High-Grade E**

Masahito Kawazu^1,2^, Ayumi Taguchi^3^, Emiko Yoshida^4,5^, Hiroshi Yoshida^6^, Masaya Uno^7^, Satoshi Inoue^2,8^, Yoko Yamamoto^3^, Shingo Sakashita^9^, Toshihide Ueno^2^, Yuki Nakamura^1^, Jason Lin^1^, Shinya Kojima^2^, Katsushige Kawase^1,10^, Aya Ishizaka^3^, Suguru Miyata^1,10^, Motohiro Kojima^9^, Masako Ikemura^11^, Kenbun Sone^3^, Mitsuya Ishikawa^7^, Tomoyasu Kato^7^, Hiroyuki Mano^2^, Yasuhisa Terao^4^, Katsutoshi Oda^12^

1 Division of Cell Therapy, Chiba Cancer Center Research Institute

2 Division of Cellular Signaling, National Cancer Center Research Institute

3 Department of Gynecology, Graduate School of Medicine, The University of Tokyo

4 Department of Gynecology, Graduate School of Medicine, Juntendo University

5 Diagnosis and Therapeutics of Intractable Diseases and Intractable Disease Research Center, Juntendo University Graduate School of Medicine

6 Department of Diagnostic Pathology, National Cancer Center Hospital

7 Department of Gynecology, National Cancer Center Hospital

8 Division of Tumor Immunology, Institute for Advanced Medical Research, Keio University School of Medicine

9 Division of Pathology, Exploratory Oncology Research & Clinical Trial Center, National Cancer Center Japan

10 Department of Otorhinolaryngology/Head & Neck Surgery, Graduate School of Medicine, Chiba University

11 Department of Pathology, Graduate School of Medicine, The University of Tokyo

12 Division of Integrative Genomics, Graduate School of Medicine, The University of Tokyo

**Mutational profile of high-grade endometrial carcinomas**

The median number of mutations per Mbp was 169 for POLE tumors, 20.7 for MSI-H tumors, 1.27 for TP53 tumors, 1.27 for NSMP tumors, and 18.5 for grade 1–2 MSI-H tumors (Figure 1A). Mutations in *PTEN* and *ARID1A* were frequently observed in POLE and MSI-H tumors (Figures 1B, 1C). Germline mutations in MMR genes were identified in three MSI-H tumors (one in *MLH1* and two in *MSH6*).

Although somatic mutations in *BRCA1* and *BRCA2* were frequently observed (12% and 16%, respectively, Table S3), most of these mutations were found in *POLE* or MSI-H tumors, suggesting that their contribution to chromosomal instability is likely limited. Germline mutations in *BRCA1*/*2* genes were not identified. In contrast to previous result^1^, *KRAS* mutations were more common in NSMP tumors (30%) than in the other tumor types (4.9%) (Figures 1B, 1C).

Analysis of base substitution patterns revealed that the *POLE* type is characterized by the dominant *POLE* signature. In *POLE* tumors, mutations in mismatch repair (MMR) genes were sporadically observed; those tumors have both the *POLE* signature and the MMRd signature (Figures 1A, Supplementary Figure S4). It is likely that the accumulation of gene mutations due to *POLE* mutations affects MMR genes, leading to the acquisition of MSI-H characteristics. It is likely that *POLE* tumors become MMRd at relatively later stages of cancer development, with potentially different MMRd status than those observed in MSI-H tumors that occur in the initial phase of cancer development. Of note, two possible *POLE* tumors, which carried mutations in *POLD1* and the *POLD1* mutational signature, were by definition categorized as MSI-H tumors (Figures 1A, S4). The most predominant mutational signature in *TP53*-mut and NSMP tumors was the age signature, followed by the apolipoprotein B mRNA editing enzyme, catalytic polypeptide (APOBEC) signature (Figures 1A, S4).

The pattern of CN abnormalities^2^ revealed that *TP53*-mut tumors had a notable prevalence of the CN signature related to homologous recombination deficiency (HRD), especially CN signature 18 (CN18) (Figures 1A, 3C). The frequency of CN17, typically associated with HRD tumors in high-grade serous ovarian cancer and triple-negative breast cancer^2^, was not notably high (Figure 3C). Given reported correlations between CN18 or CN19 and homologous recombination repair defects coupled with two genome doubling events, high-level accumulation of chromosomal abnormalities associated with *TP53* mutations in high-grade EC might contribute to the CN18 signature.

The scarcity of mutations in *CTNNB1*, which were reported to be common in NSMP and CN-L tumors^3^, might be associated with the high-grade nature of tumors in this study.

**Discordance in subtype classification**

Discordance between *TP53* mutation call, p53 IHC staining, and CN-based molecular classification was prominent in this cohort (Figure 2A, Supplementary Table S1). In examining discordant cases, three principal sources of disagreement emerged: (i) false-negative detection by either IHC or WES; (ii) the intrinsic challenge of setting a robust cutoff for the extent of chromosomal instability; and (iii) chromosomal instability arising from mechanisms independent of TP53 abnormalities.

Discordance between genomic and IHC subtypes was observed in 8 of 55 non-POLE/non-MSI cases. Reported discordance rates range from approximately 6–7% among non-hypermutant TP53-mutated cases, frequently involving hotspot missense or splice-site mutations^4,5^. Detailed re-evaluation of our data indicated that most discrepancies arose from false-negative *TP53* mutation calls by WES, which were attributable to low variant allele frequency, or inconsistent detection among the three informatics tools used in our pipeline (Supplementary Table S5). Conversely, false-negative p53 IHC might occur in cases with attenuated staining possibly due to technical causes (e.g., suboptimal fixation or age-related degradation of the specimen).

Discrepancies between CN-based classification and *TP53*/p53 status were found in 16 cases, indicating that p53-independent mechanisms can also drive CN alterations, particularly in high-grade ECs. It should be noted that standard CN-Low tumors (typically low-grade endometrioid carcinomas) are absent from this high-grade cohort, which may relatively enrich for NSMP tumors with intermediate or higher genomic instability. In addition, several *TP53*-aberrant tumors exhibited clear chromosomal instability that nevertheless remained below the predetermined cutoff (Supplementary Table S5, Supplementary Figure S11); these were classified as CN-L, underscoring the inherent difficulties in quantitative distinction between CN-L and CN-H. We also noticed that CN alterations sometimes failed to pass our filter due to low cancer cell fraction, resulting in the false-negative call. Collectively, these findings support the notion that the current molecular subtyping strategies remain to be improved or complemented by additional stratification axis.

**Case-level interpretation of *TP53* classification discordance**

To clarify the mechanisms underlying discordance among *TP53* mutation status by WES, p53 immunohistochemistry, and copy-number–based classification, we performed detailed case-level analyses (Supplementary Tables S5–S8).

For cases lacking detectable *TP53* mutations by WES but showing p53-null IHC with copy-number alterations (ID-53, ID-60, ID-77), these findings are consistent with genetic events that are difficult to detect by WES, such as large deletions or other structural alterations. In ID-16, the presence of two variant allele frequencies without LOH suggests biallelic loss-of-function mutations, which is not necessarily associated with discordance (Supplementary Table S7). For ID-47, the wild-type p53 immunohistochemistry pattern and the absence of clear loss of heterozygosity do not exclude the possibility that p53 function is preserved. However, alternative explanations cannot be excluded, including potential misclassification of the copy-number status (AAABB) or failure to detect a second *TP53* alteration affecting the contralateral allele. Given the relatively high variant allele frequency (57%), we preferentially interpret this case as copy-number–high driven by *TP53* alteration rather than true functional preservation. For ID-58, retention of the contralateral allele may indicate potential preservation of p53 function despite p53 overexpression. Thus, misclassification based on WES cannot be excluded in situations where a mutation affecting only one TP53 allele is not sufficient to alter p53 function or p53 IHC patterns.

With respect to *TP53* missense mutations reported to function as dominant-negative (DN) or gain-of-function (GOF), we note that many well-known hotspot variants have been described as exhibiting DN and/or GOF properties (IARC the TP53 database, https://tp53.cancer.gov/). Indeed, such variants were observed in discordant cases, including a heterozygous case, in our cohort (e.g., p.Pro152Leu (ID-58), p.Arg175His (ID-20), p.Arg248Trp (ID-50), p.Arg282Trp (ID-62)); however, they were also observed in cases with loss of heterozygosity as well as in fully concordant cases. For example, p.Pro152Leu, which has been reported to exhibit DN or GOF activity, was observed in ID-67 (loss of heterozygosity (AAA status), fully concordant). Thus, it is challenging to assess the effect of DN/GOF-associated *TP53* missense mutations in this cohort of limited number of cases.

Consistent with this case-level assessment, summary analyses (Supplementary Tables S6–S8) did not reveal enrichment of specific mutation types or allelic configurations in discordant cases. Instead, discordance was more frequently associated with cases lacking detectable *TP53* mutations by WES or showing p53-null IHC, suggesting contributions from genetic events that are difficult to detect by WES (e.g., large deletions or structural alterations), as well as from CN-H states arising through mechanisms independent of TP53 mutation. Taken together, these findings indicate that TP53 classification discordance in this cohort is more likely driven by biological heterogeneity in underlying molecular mechanisms and technical limitations of current detection and classification approaches, underscoring the inherent challenge in the precise discrimination between cases with CN-H and CN-L in high-grade ECs.

**Gene signatures scores representing transcriptome clusters**

To generalize the clustering procedure described above, we selected 8 genes considered to represent the glandular/luminal phenotype, 11 genes considered to represent the ciliated phenotype, and 7 genes considered to represent the carcinosarcoma phenotype. We performed ssGSEA to calculate the score of each signature (Table S5). Signature scores consistent with the clustering results were obtained (Figure 4F). Endometrioid tumors (EMG3s) exhibited a strong ciliated phenotype, clear cell carcinomas exhibited a strong glandular/luminal phenotype, and serous tumors exhibited a moderate but apparent glandular/luminal phenotype (Figure 4G).

**Validation of clustering with selected genes and the TCGA dataset**

To validate our analysis, we analyzed TCGA data obtained from University of California, Santa Cruz (UCSC) Xena^6^. Clustering using selected 96 relevant genes (Table S6) via t-SNE revealed three tumor clusters and one normal cluster (Supplementary Figure S6). Similar to the clustering of high-grade EC in this study, Cluster T1 was characterized by high expression of genes associated with glandular/luminal cells. Cluster T2 was characterized by high expression of genes associated with ciliated cells (Supplementary Figures S6, S7). Cluster T3 was mainly composed of carcinosarcomas. Cluster T1 was mainly composed of serous carcinomas, possibly due to the lack of clear cell carcinomas in the TCGA dataset. Despite this difference, which is discussed in detail in the supplementary materials, the results indicate that marker gene–based classification is also feasible within the TCGA dataset.


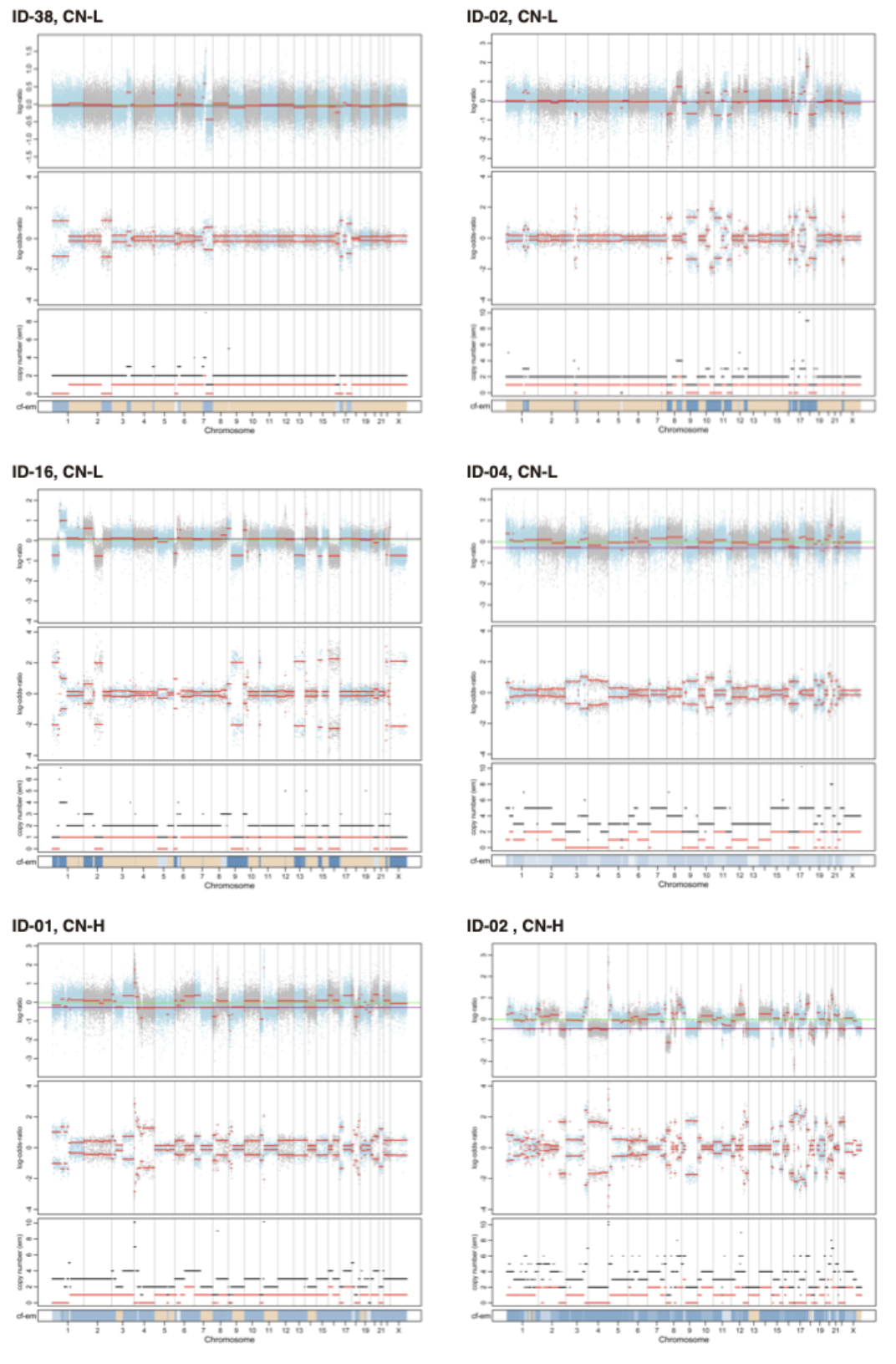


**Supplementary Figure S11. Varied levels of chromosomal instability across samples.**Representative examples of allele-specific copy number profiles of high-grade endometrial carcinoma cases, determined using FACETS, are shown. From top to bottom, panels display log2 tumor-to-normal copy ratio, allelic imbalance (log odds ratio), and integer copy number estimates across the genome. Black lines indicate total copy number, while red lines indicate minor allele copy number; chromosomes are shown along the x-axis. These examples illustrate that the extent of chromosomal instability varies across tumors, and that cases with appreciable copy number alterations may still be classified as CN-L when the proportion of the genome affected remains below a predetermined cutoff.

**References**

1 Levine DA. Integrated genomic characterization of endometrial carcinoma. *Nature* 2013; 497: 67–73.

2 Steele CD, Abbasi A, Islam SMA, Bowes AL, Khandekar A, Haase K *et al.* Signatures of copy number alterations in human cancer. *Nature* 2022; 606: 984–991.

3 Stelloo E, Nout RA, Osse EM, Jürgenliemk-Schulz IJ, Jobsen JJ, Lutgens LC *et al.* Improved Risk Assessment by Integrating Molecular and Clinicopathological Factors in Early-stage Endometrial Cancer—Combined Analysis of the PORTEC Cohorts. *Clinical Cancer Research* 2016; 22: 4215–4224.

4 Momeni-Boroujeni A, Dahoud W, Vanderbilt CM, Chiang S, Murali R, Rios-Doria E V *et al.* Clinicopathologic and Genomic Analysis of TP53-Mutated Endometrial Carcinomas. *Clin Cancer Res* 2021; 27: 2613–2623.

5 Rios-Doria E, Momeni-Boroujeni A, Friedman CF, Selenica P, Zhou Q, Wu M *et al.* Integration of clinical sequencing and immunohistochemistry for the molecular classification of endometrial carcinoma. *Gynecol Oncol* 2023; 174: 262–272.

6 Goldman MJ, Craft B, Hastie M, Repečka K, McDade F, Kamath A *et al.* Visualizing and interpreting cancer genomics data via the Xena platform. *Nat Biotechnol* 2020; 38: 675–678.
