# Supplementary material for: Genomic and Transcriptomic Analysis of High-Grade Endometrial Carcinoma Reveals Biological Heterogeneity and Molecular Classification Challenges
Source: Cancer Res Commun. 2026 Apr 28;6(4):961–75. doi: 10.1158/2767-9764.CRC-25-0589 (PMC13123251; doi:10.1158/2767-9764.CRC-25-0589)
Supplement: Supplementary Figure S1 — Workflow of sample processing and analytical steps. [file crc-25-0589_supplementary_figure_s1_suppsf1.docx]

**
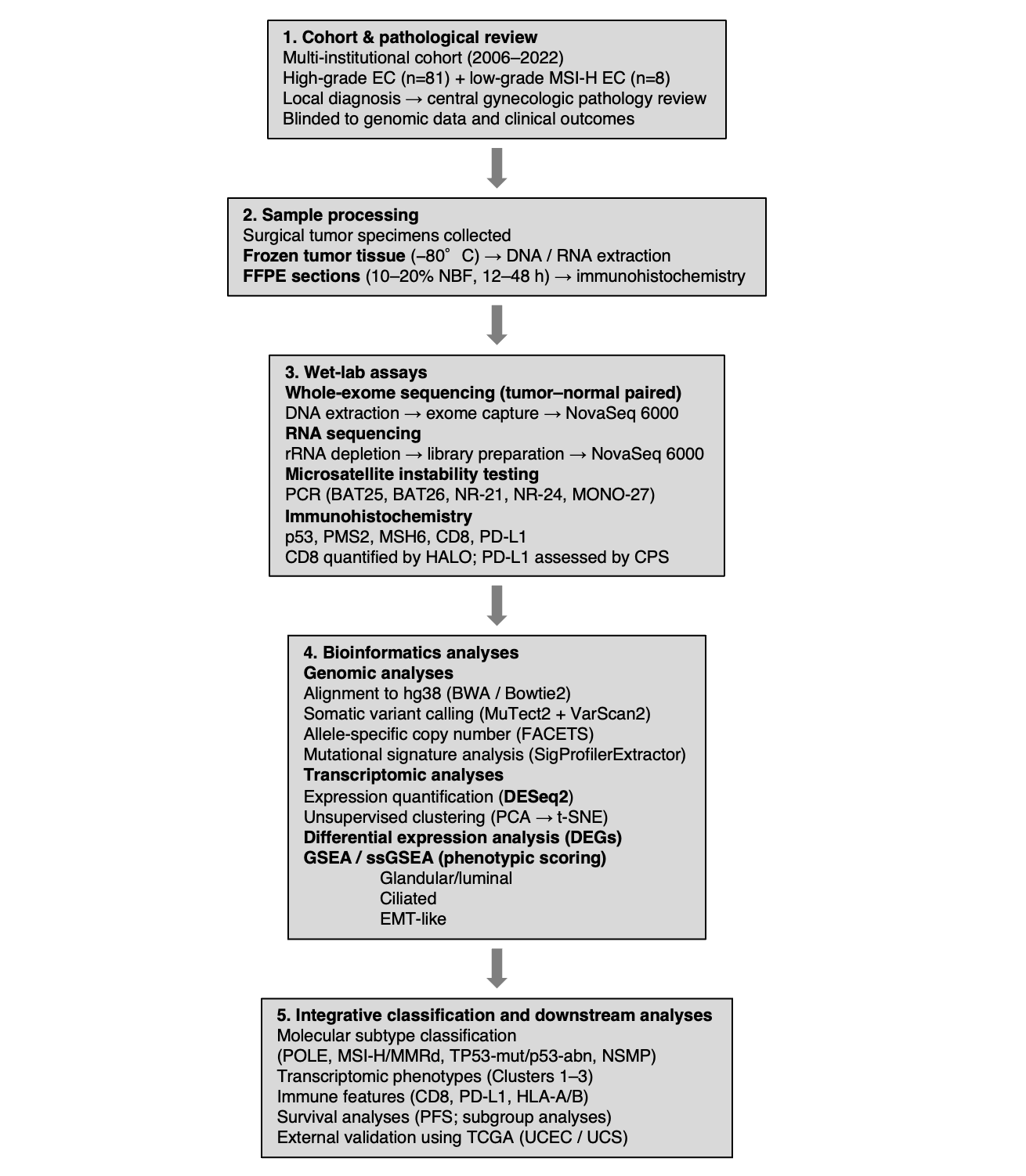
**

**Supplementary Figure S1. Workflow of sample processing and analytical steps.**

Tumor specimens obtained at surgery were processed according to routine clinical practice and research protocols. Formalin-fixed paraffin-embedded (FFPE) sections were prepared and archived as part of standard pathological diagnosis, whereas frozen tumor tissues were obtained from residual surgical material and stored specifically for research purposes. Tumor–normal paired whole-exome sequencing, RNA sequencing, microsatellite instability testing, and immunohistochemistry were performed. Sequencing data were analyzed to identify somatic variants, allele-specific copy-number alterations, and mutational signatures. RNA-seq data were quantified using DESeq2, followed by unsupervised clustering, differential expression analysis, and gene set enrichment analyses to derive transcriptomic phenotypes. Molecular and transcriptomic classifications were integrated with immune features and clinical outcomes, with external validation using TCGA datasets.
