# Supplementary material for: Genomic and Transcriptomic Analysis of High-Grade Endometrial Carcinoma Reveals Biological Heterogeneity and Molecular Classification Challenges
Source: Cancer Res Commun. 2026 Apr 28;6(4):961–75. doi: 10.1158/2767-9764.CRC-25-0589 (PMC13123251; doi:10.1158/2767-9764.CRC-25-0589)
Supplement: Supplementary Figure S2 — Histology of high-grade endometrial carcinomas. [file crc-25-0589_supplementary_figure_s2_suppsf2.docx]

**
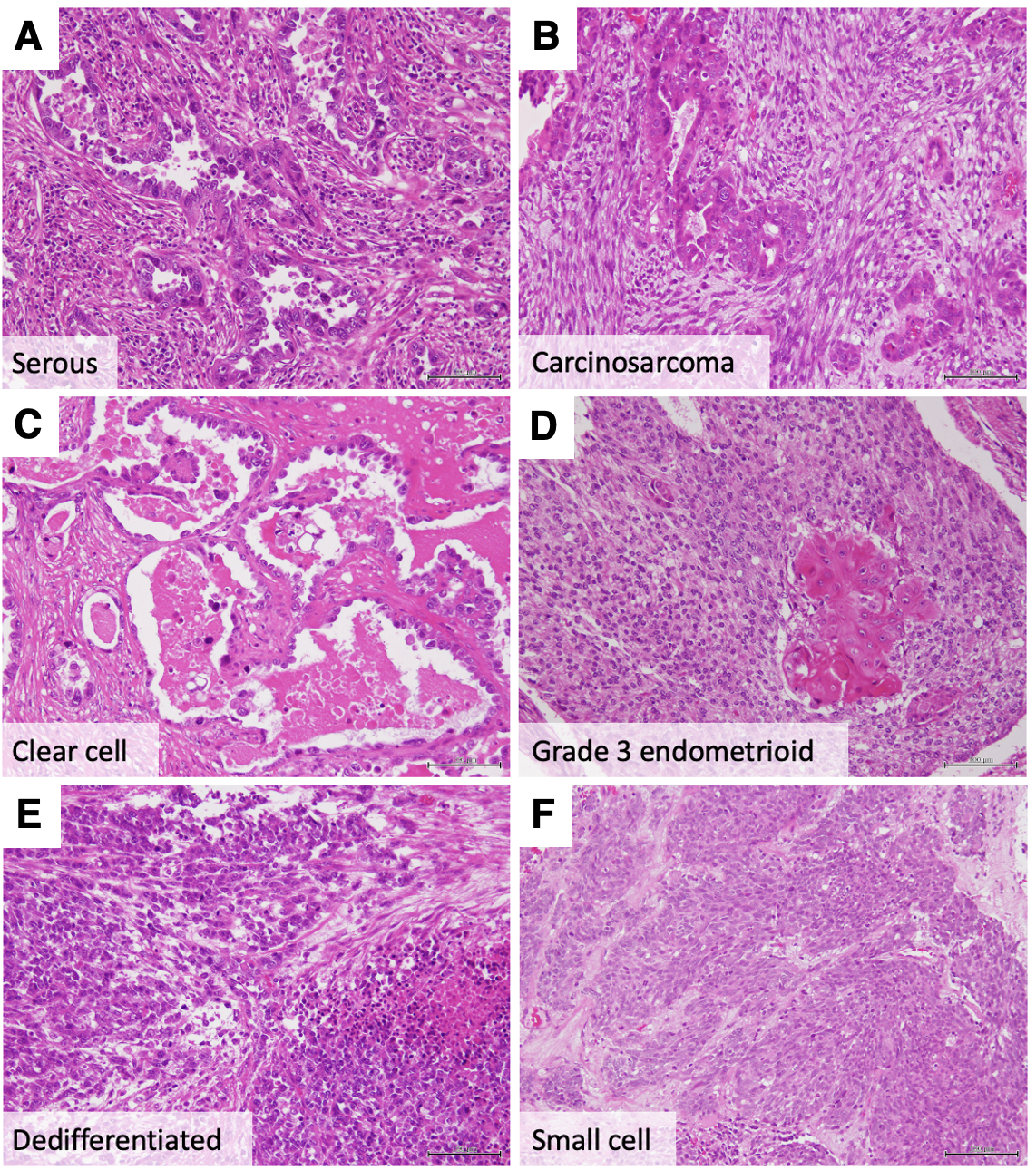
**

**Supplementary Figure S2. Histology of high-grade endometrial carcinomas.**

1. Serous carcinoma. Tumor cells with marked nuclear atypia have irregularly shaped glands.
2. Carcinosarcoma. Both high-grade adenocarcinoma components resembling serous carcinoma and sarcomatous components composed of pleomorphic spindle cells are observed.
3. Clear cell carcinoma. Cuboidal tumor cells with clear to eosinophilic cytoplasm have a tubulocystic pattern and papillary structures.
4. Grade 3 endometrioid carcinoma. Solid tumor nests with focal squamous differentiation are observed.
5. Dedifferentiated carcinoma. Undifferentiated carcinoma components are composed of monotonous discohesive tumor cells.
6. Small cell carcinoma. Tumor cells with enlarged nuclei are arranged in solid nests. These tumor cells express neuroendocrine markers.

**A**–**F**, hematoxylin and eosin stain, ×200, scale bars indicate 100 μm
