# Supplementary material for: Genomic and Transcriptomic Analysis of High-Grade Endometrial Carcinoma Reveals Biological Heterogeneity and Molecular Classification Challenges
Source: Cancer Res Commun. 2026 Apr 28;6(4):961–75. doi: 10.1158/2767-9764.CRC-25-0589 (PMC13123251; doi:10.1158/2767-9764.CRC-25-0589)
Supplement: Supplementary Figure S3 — Representative results of immunohistochemical staining. [file crc-25-0589_supplementary_figure_s3_suppsf3.docx]

**
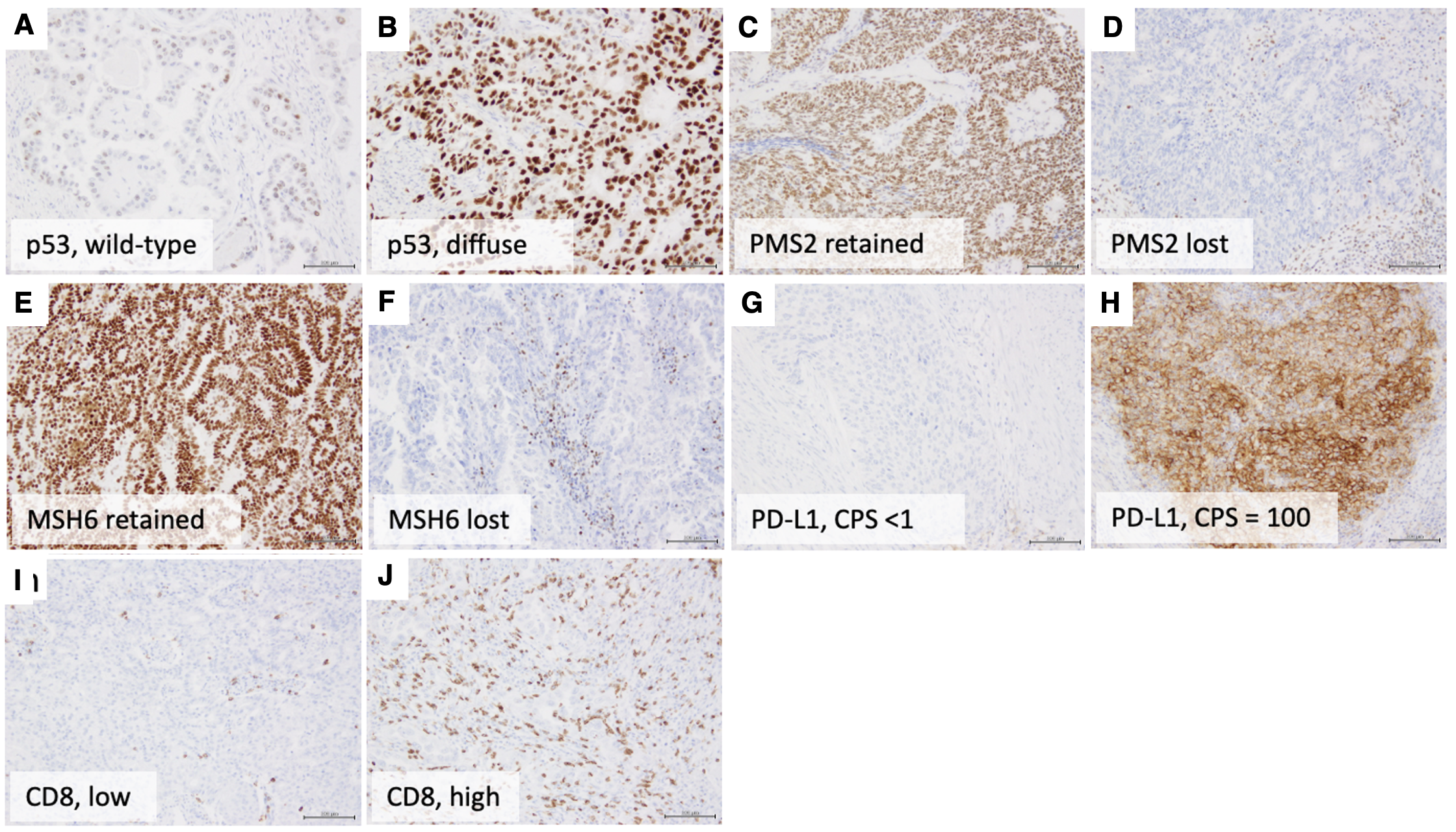
**

**Supplementary Figure S3. Representative results of immunohistochemical staining.**

1. p53 wild-type staining pattern.
2. Diffuse strong p53 positivity in tumor cells, indicating TP53 gene alteration.
3. Retained PMS2 expression in tumor and normal stromal cells.
4. PMS2 expression is lost only in tumor cells.
5. Retained MSH6 expression in tumor and normal stromal cells.
6. MSH6 expression is lost only in tumor cells.
7. This tumor has a combined positive score (CPS) of less than 1.
8. Another tumor with a CPS of 100.
9. Scattered peritumoral CD8+ T cells are observed.
10. Numerous CD8+ T cells are seen in another tumor.

**A**–**J**, ×200, scale bars indicate 100 μm
