# Supplementary material for: Genomic and Transcriptomic Analysis of High-Grade Endometrial Carcinoma Reveals Biological Heterogeneity and Molecular Classification Challenges
Source: Cancer Res Commun. 2026 Apr 28;6(4):961–75. doi: 10.1158/2767-9764.CRC-25-0589 (PMC13123251; doi:10.1158/2767-9764.CRC-25-0589)
Supplement: Supplementary Figure S4 — Microsatellite instability score and mutational signatures for each sample across different molecular subtypes. [file crc-25-0589_supplementary_figure_s4_suppsf4.docx]

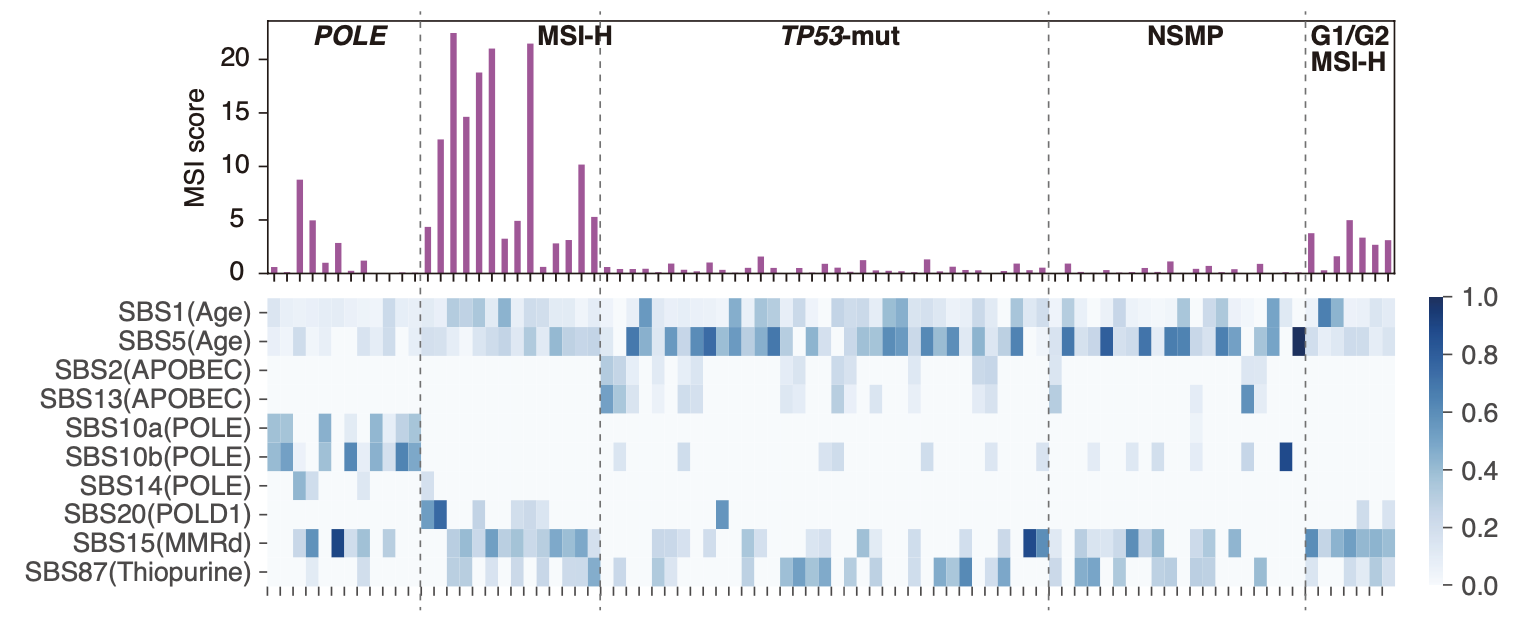


**Supplementary Figure S4. Microsatellite instability score and mutational signatures for each sample across different molecular subtypes.**

The top panel shows the MSI score. The bottom panel shows the relative contribution of different single base substitution signatures.

APOBEC, apolipoprotein B mRNA editing enzyme, catalytic polypeptide; G1/G2, grade 1–2 endometrioid; MMRd, mismatch repair deficient; MSI-H, microsatellite instability–high; NSMP, no specific molecular profile; SBS, single base substitution.
