# Supplementary material for: Genomic and Transcriptomic Analysis of High-Grade Endometrial Carcinoma Reveals Biological Heterogeneity and Molecular Classification Challenges
Source: Cancer Res Commun. 2026 Apr 28;6(4):961–75. doi: 10.1158/2767-9764.CRC-25-0589 (PMC13123251; doi:10.1158/2767-9764.CRC-25-0589)
Supplement: Supplementary Figure S6 — Clustering analysis based on gene expression in the TCGA dataset. [file crc-25-0589_supplementary_figure_s6_suppsf6.docx]

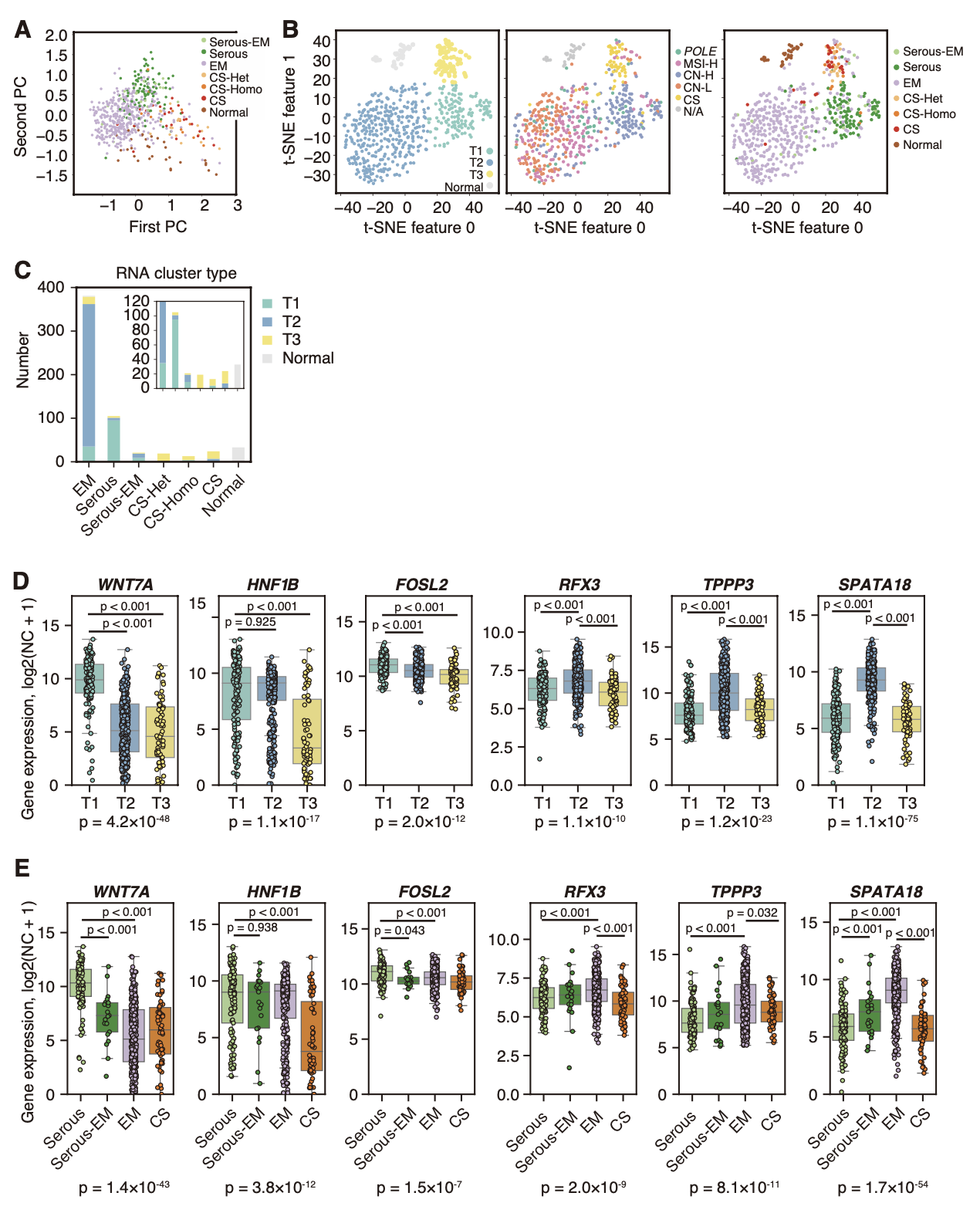


**Supplementary Figure S6. Clustering analysis based on gene expression in the TCGA dataset.**

1. Principal component analysis plot showing the distribution of samples in the TCGA dataset based on gene expression profile.
2. The left t-SNE plot shows the results of spectral clustering, indicating the RNA cluster classification in the TCGA dataset (T1, T2, T3, Normal). The middle t-SNE plot shows the distribution of samples across different molecular subtypes. The right t-SNE plot shows the distribution of samples across different histological types.
3. Bar plots showing the number of samples of each histological type across RNA clusters (T1, T2, T3, Normal). The inset plot adjusts the y-axis to highlight areas with smaller values, making it easier to visualize the differences in the distribution of histological types with fewer samples.
4. Box plots showing the expression levels of representative genes across different RNA clusters (T1, T2, T3) in the TCGA dataset. The y-axis represents the log2(normalized count + 1) of gene expression. P-values are provided for comparisons using ANOVA followed by Tukey’s post hoc analysis.
5. Box plots showing the expression levels of representative genes across different histological subtypes in the TCGA dataset limited to endometroid carcinoma. The y-axis represents the log_2_(normalized count + 1) of gene expression. P-values are provided for comparisons using ANOVA followed by Tukey’s post hoc analysis.

ANOVA, analysis of variance; Clear-EM, clear cell carcinoma mixed with endometrioid endometrial carcinoma; Clear-S, clear cell carcinoma mixed with serous carcinoma; CS, carcinosarcoma; EMG3, grade 3 endometroid; G1/G2, grade 1–2 endometroid; MSI-H, microsatellite instability–high; N/A, not available; PC, principal component; TCGA, The Cancer Genome Atlas program; t-SNE, t-distributed stochastic neighbor embedding.
