# Supplementary material for: Genomic and Transcriptomic Analysis of High-Grade Endometrial Carcinoma Reveals Biological Heterogeneity and Molecular Classification Challenges
Source: Cancer Res Commun. 2026 Apr 28;6(4):961–75. doi: 10.1158/2767-9764.CRC-25-0589 (PMC13123251; doi:10.1158/2767-9764.CRC-25-0589)
Supplement: Supplementary Figure S7 — Gene expression profiles across different RNA clusters in The Cancer Genome Atlas Program dataset. [file crc-25-0589_supplementary_figure_s7_suppsf7.docx]

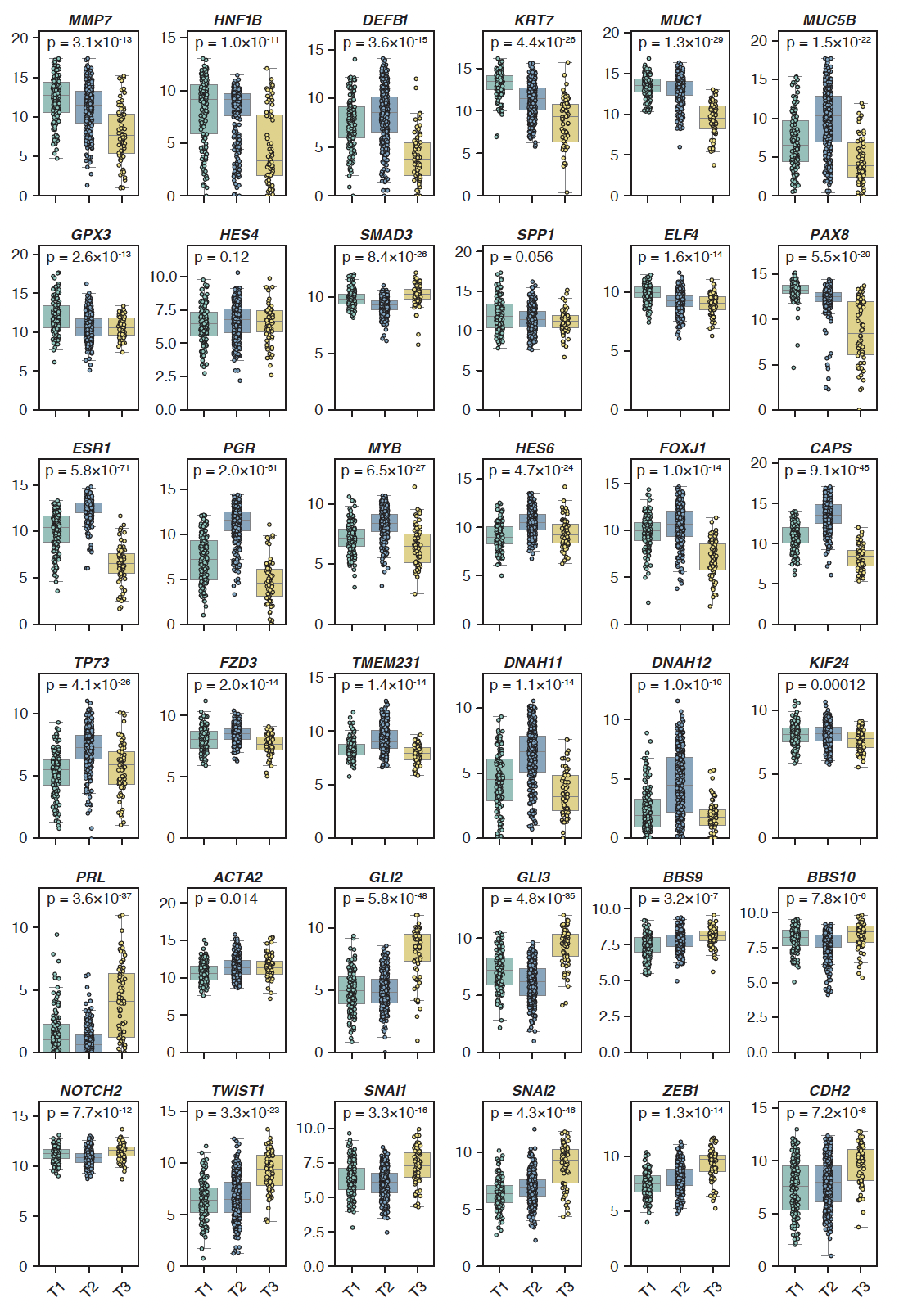


**Supplementary Figure S7. Gene expression profiles across different RNA clusters in The Cancer Genome Atlas Program dataset.**

Box plots showing the expression levels of various genes across different RNA clusters in The Cancer Genome Atlas Program dataset. The y-axis represents the log_2_(normalized count + 1) of gene expression. Analysis of variance p-values are provided.
