# Supplementary material for: Genomic and Transcriptomic Analysis of High-Grade Endometrial Carcinoma Reveals Biological Heterogeneity and Molecular Classification Challenges
Source: Cancer Res Commun. 2026 Apr 28;6(4):961–75. doi: 10.1158/2767-9764.CRC-25-0589 (PMC13123251; doi:10.1158/2767-9764.CRC-25-0589)
Supplement: Supplementary Figure S8 — Immunological features of tumors according to phenotypic status. [file crc-25-0589_supplementary_figure_s8_suppsf8.docx]

**
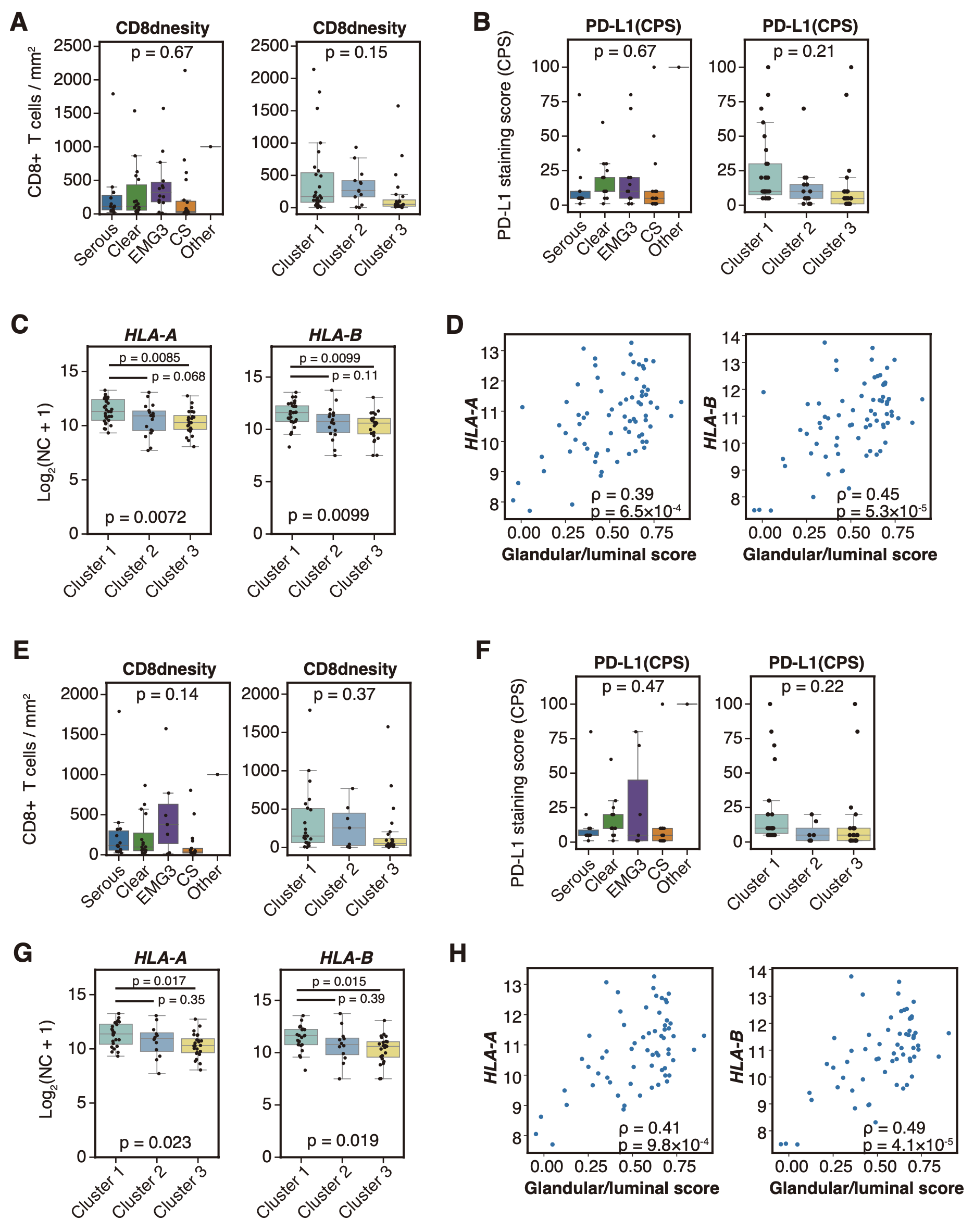
**

**Supplementary Figure S8. Immunological features of tumors according to phenotypic status.**(**A**, **E**) Box plots showing the density of CD8+ T cells across different histological subtypes and RNA clusters, after exclusion of MSI-H tumors (**A**) or exclusion of MSI-H and POLE-mutated tumors (**E**).

(**B**, **F**) Box plots showing the PD-L1 staining across different histological subtypes and RNA clusters, after exclusion of MSI-H tumors (**B**) or exclusion of MSI-H and POLE-mutated tumors (**F**).

(**C**, **G**) Box plots showing the expression of HLA-A and HLA-B across different RNA clusters, after exclusion of MSI-H tumors (**C**) or exclusion of MSI-H and POLE-mutated tumors (**G**).

(**D**, **H**) Scatter plots showing the correlation between glandular/luminal score and the expression of HLA-A and HLA-B, after exclusion of MSI-H tumors (**D**) or exclusion of MSI-H and POLE-mutated tumors (**H**). Spearman’s rank correlation coefficients and p-values are indicated.
