# Supplementary material for: Genomic and Transcriptomic Analysis of High-Grade Endometrial Carcinoma Reveals Biological Heterogeneity and Molecular Classification Challenges
Source: Cancer Res Commun. 2026 Apr 28;6(4):961–75. doi: 10.1158/2767-9764.CRC-25-0589 (PMC13123251; doi:10.1158/2767-9764.CRC-25-0589)
Supplement: Supplementary Figure S9 — Analysis of immunological status in the TCGA dataset. [file crc-25-0589_supplementary_figure_s9_suppsf9.docx]

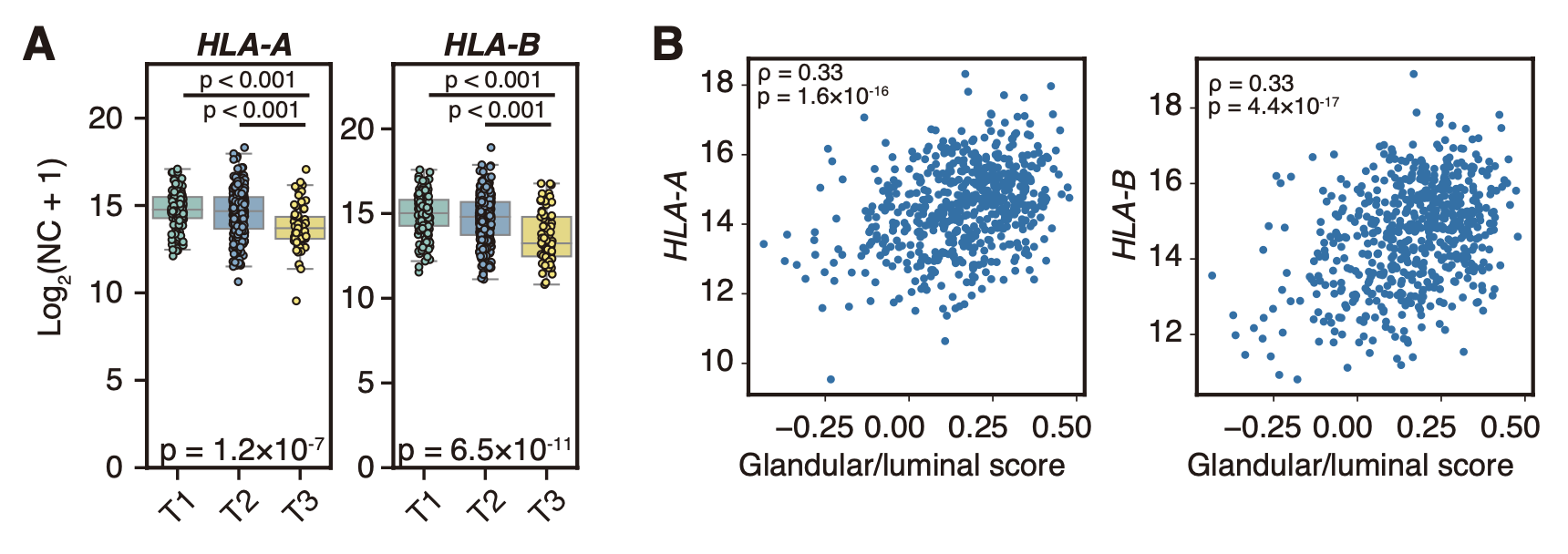


**Supplementary Figure S9. Analysis of immunological status in the TCGA dataset.**

1. Box plots showing the expression of *HLA-A* and *HLA-B* across different tumor clusters in the TCGA dataset.
2. Scatter plots showing the correlation between glandular/luminal score and the expression of *HLA-A* and *HLA-B* in this study cohort and in the TCGA dataset. Spearman’s rank correlation coefficients and p-values are indicated.
