# Supplementary material for: Genomic and Transcriptomic Analysis of High-Grade Endometrial Carcinoma Reveals Biological Heterogeneity and Molecular Classification Challenges
Source: Cancer Res Commun. 2026 Apr 28;6(4):961–75. doi: 10.1158/2767-9764.CRC-25-0589 (PMC13123251; doi:10.1158/2767-9764.CRC-25-0589)
Supplement: Supplementary Figure S10 — Prognostic impact of transcriptomic clusters in TP53-mutated, high-grade endometrial carcinoma. [file crc-25-0589_supplementary_figure_s10_suppsf10.docx]

**
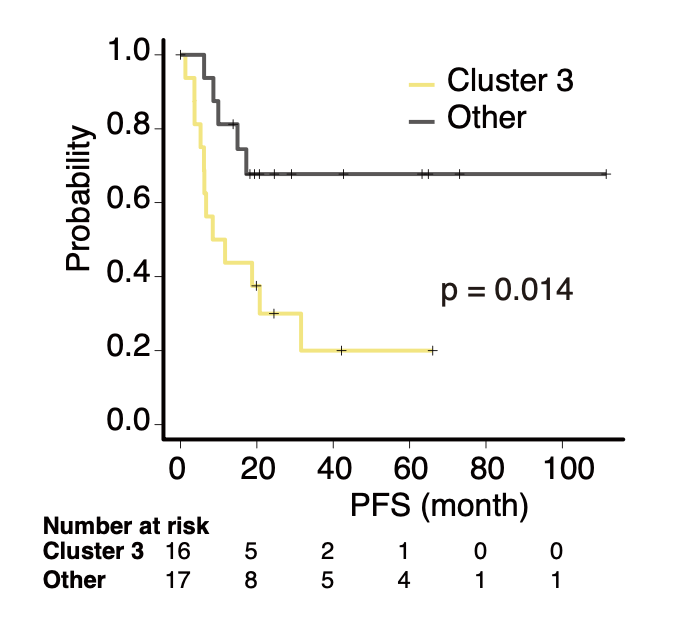
**

**Supplementary Figure S10. Prognostic impact of transcriptomic clusters in *TP53*-mutated, high-grade endometrial carcinoma.**

Kaplan–Meier curves for progression-free survival (PFS) according to RNA clusters in the molecular subtype of *TP53*-mut (n=33).
